# Author Correction: Genetic investigation of fibromuscular dysplasia identifies risk loci and shared genetics with common cardiovascular diseases

**DOI:** 10.1038/s41467-022-29921-1

**Published:** 2022-04-20

**Authors:** Adrien Georges, Min-Lee Yang, Takiy-Eddine Berrandou, Mark K. Bakker, Ozan Dikilitas, Soto Romuald Kiando, Lijiang Ma, Benjamin A. Satterfield, Sebanti Sengupta, Mengyao Yu, Jean-François Deleuze, Delia Dupré, Kristina L. Hunker, Sergiy Kyryachenko, Lu Liu, Ines Sayoud-Sadeg, Laurence Amar, Chad M. Brummett, Dawn M. Coleman, Valentina d’Escamard, Peter de Leeuw, Natalia Fendrikova-Mahlay, Daniella Kadian-Dodov, Jun Z. Li, Aurélien Lorthioir, Marco Pappaccogli, Aleksander Prejbisz, Witold Smigielski, James C. Stanley, Matthew Zawistowski, Xiang Zhou, Sebastian Zöllner, FEIRI investigators, Peter de Leeuw, International Stroke Genetics Consortium (ISGC) Intracranial Aneurysm Working Group, MEGASTROKE, Philippe Amouyel, Marc L. De Buyzere, Stéphanie Debette, Piotr Dobrowolski, Wojciech Drygas, Heather L. Gornik, Jeffrey W. Olin, Jerzy Piwonski, Ernst R. Rietzschel, Ynte M. Ruigrok, Miikka Vikkula, Ewa Warchol Celinska, Andrzej Januszewicz, Iftikhar J. Kullo, Michel Azizi, ARCADIA Investigators, Xavier Jeunemaitre, Alexandre Persu, Jason C. Kovacic, Santhi K. Ganesh, Nabila Bouatia-Naji

**Affiliations:** 1 PARCC, INSERM, Université de Paris, F-750015 Paris, France; 2 Division of Cardiovascular Medicine, Department of Internal Medicine, University of Michigan Medical School, Ann Arbor, MI USA; 3 Department of Human Genetics, University of Michigan Medical School, Ann Arbor, MI USA; 4 Department of Neurology and Neurosurgery, University Medical Center Utrecht Brain Center, Utrecht University, Utrecht, the Netherlands; 5 Department of Cardiovascular Medicine, Mayo Clinic, Rochester, MN 55902 USA; 6 Icahn Institute for Genomics and Multiscale Biology, Icahn School of Medicine at Mount Sinai, New York, NY USA; 7 Centre National de Recherche en Génomique Humaine, Institut de Génomique, CEA and Fondation Jean Dausset-CEPH, Evry, France; 8 Hypertension Unit, Assistance Publique-Hôpitaux de Paris, Hôpital Européen Georges Pompidou, F-75015 Paris, France; 9 Department of Anesthesiology, Michigan Medicine, University of Michigan, Ann Arbor, MI USA; 10 Vascular Surgery Section, Department of Surgery, Michigan Medicine, University of Michigan, Ann Arbor, MI 48109 USA; 11 Cardiovascular Institute, Icahn School of Medicine at Mount Sinai, New York, NY USA; 12 Department of Internal Medicine, Division of General Internal Medicine, Section Vascular Medicine, Maastricht University Medical Centre, Maastricht University, Maastricht, the Netherlands; 13 CARIM School for Cardiovascular Diseases, Maastricht University Medical Centre, Maastricht University, Maastricht, the Netherlands; 14 Heart and Vascular Institute, Cleveland Clinic, Cleveland, OH 44195 USA; 15 Zena and Michael A. Wiener Cardiovascular Institute and Marie-Josée and Henry R, Kravis Center for Cardiovascular Health Icahn School of Medicine at Mount Sinai, New York, NY USA; 16 Division of Cardiology, Cliniques Universitaires Saint-Luc, Université Catholique de Louvain, 1200 Brussels, Belgium; 17 Division of Internal Medicine and Hypertension Unit, Department of Medical Sciences, University of Turin, Turin, Italy; 18 Department of Hypertension, National Institute of Cardiology, Warsaw, Poland; 19 Department of Demography, University of Lodz, Lodz, Poland; 20 Department of Biostatistics and Center for Statistical Genetics, University of Michigan School of Public Health, Ann Arbor, MI USA; 21 Univ. Lille, Inserm, CHU Lille, Institut Pasteur de Lille, U1167 - RID-AGE - Labex DISTALZ - Risk Factors and Molecular Determinants of Aging-Related Disease, F-59000 Lille, France; 22 Department of Cardiovascular Diseases, Ghent University and Ghent University Hospital, Ghent, Belgium; 23 Department of Neurology, Bordeaux University Hospital, Inserm U1219, Bordeaux, France; 24 Department of Epidemiology, Cardiovascular Disease Prevention, and Health Promotion, National Institute of Cardiology, Warsaw, Poland; 25 Human Molecular Genetics, de Duve Institute, Université Catholique de Louvain, 1200 Brussels, Belgium; 26 Gonda Vascular Center, Mayo Clinic, Rochester, MN 55902 USA; 27 Université de Paris, Inserm, Centre d’Investigation Clinique 1418, F-75006 Paris, France; 28 Department of Genetics, Assistance-Publiques-Hôpitaux de Paris, Hôpital Européen Georges Pompidou, F-75015 Paris, France; 29 Pole of Cardiovascular Research, Institut de Recherche Expérimentale et Clinique, Université Catholique de Louvain, 1200 Brussels, Belgium; 30 Victor Chang Cardiac Research Institute, Darlinghurst, NSW Australia; 31 St. Vincent’s Clinical School, University of New South Wales, Sydney, NSW Australia

**Keywords:** Genome-wide association studies, Cardiovascular diseases, Genetics research

Correction to: *Nature Communications* 10.1038/s41467-021-26174-2, published online 15 October 2021.

In this article, the consortium ARCADIA Investigators was omitted. The consortium name has now been added to the author list, the list of authors who are members of the consortium (Alexandre Persu, Michel Azizi, Xavier Jeunemaitre) has been added to the paper and the full list of consortium members has been added to the Supplementary Information file. The error has been corrected in the PDF and HTML versions of the article.

## Supplementary information

Updated Supplementary Information

