# Supplementary material for: Genetic investigation of fibromuscular dysplasia identifies risk loci and shared genetics with common cardiovascular diseases
Source: Nat Commun. 2021 Oct 15;12:6031. doi: 10.1038/s41467-021-26174-2 (PMC8521585; doi:10.1038/s41467-021-26174-2)
Supplement: Supplementary file 1 — Supplementary Information [file 41467_2021_26174_MOESM1_ESM.pdf]

Supplementary Material

Genetic investigation of fibromuscular dysplasia identifies risk loci and shared genetics with common cardiovascular diseases

Georges et al.

SUPPLEMENTARY FIGURES

Figure S1. Gene-based and transcriptome wide association analyses

Quantile-quantile plot representation of SNP-based association analysis in multifocal FMD (FMD) study. $-\log_{10}$ of observed association *P*-value value (a two-sided Wald test) is represented on the y-axis, expected *P*-value on the x-axis. Genomic control value (λ_{GC}) is indicated over the graph for each study.

Figure S1

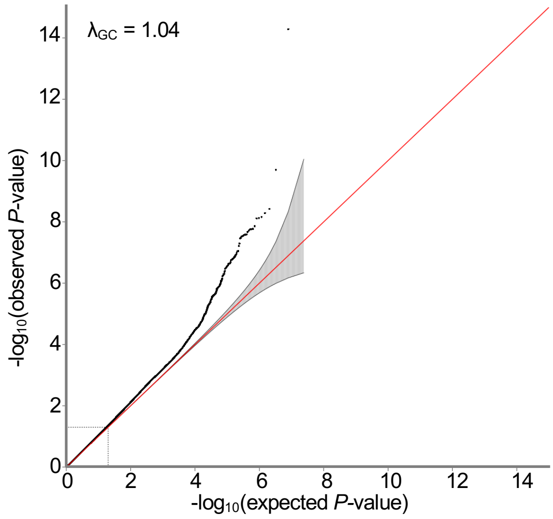

λGC = 1.04
-log10(observed P-value)
-log10(expected P-value)
0
2
4
6
8
10
12
14

Figure S2. FMD-eQTL colocalization at *ATP1B1* and *LIMA1* loci

Colocalization plot of FMD association (x-axis, log scale of *P*-value) with tibial artery (**a,d**) or aorta(**b-c**) eQTL association (y-axis, log-scale of *P*-value) at *ATP2B1* (**a**: *ATP2B1-AS1*) and *LIMA1* (**b**: *COX14*, **c**: *SMARCD1*, **d**: *LIMA1*) loci. Dot color represents the LD r^2 with the lead variant in 1000G European samples. FMD lead variant is highlighted (purple diamond). Approximate Bayes Factor Posterior Probability (*PP.abf*) for the two traits to share a common causal variant is indicated.

Figure S2

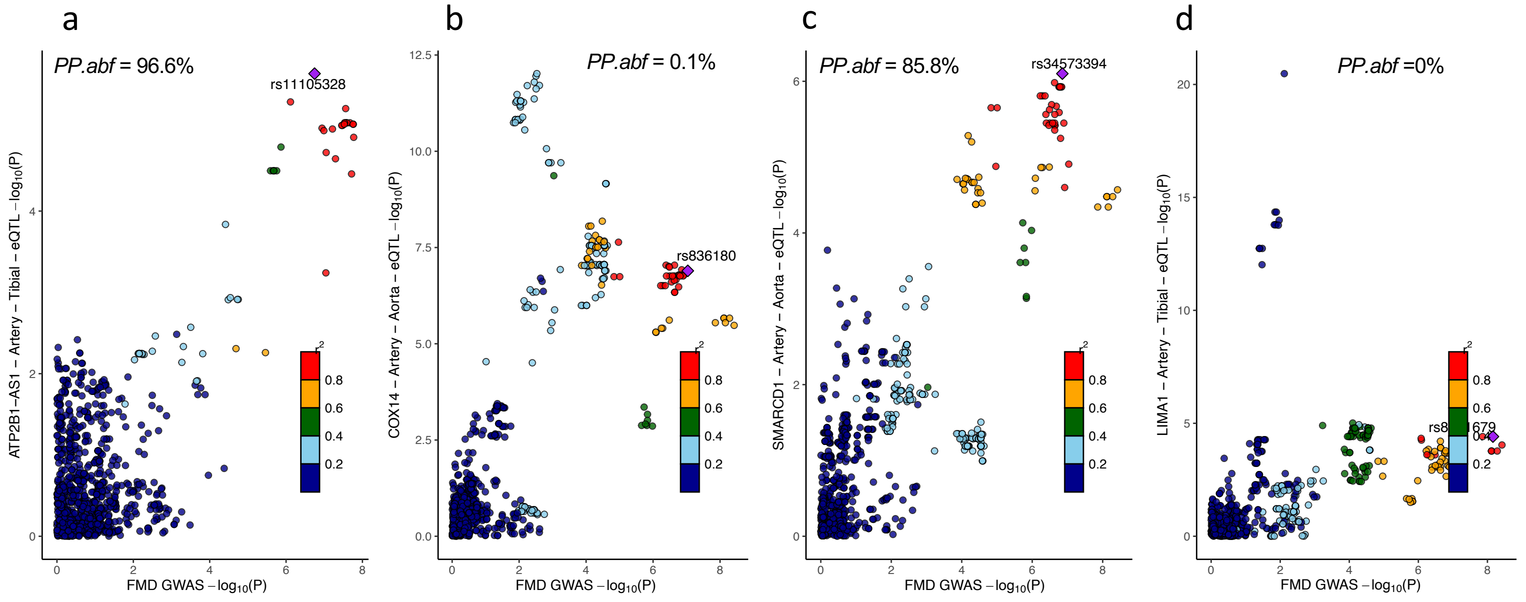

a
PP.abf = 96.6%
rs11105328
ATP2B1-AS1 − Artery − Tibial − eQTL −log10(P)
FMD GWAS −log10(P)
b
PP.abf = 0.1%
rs836180
COX14 − Artery − Aorta − eQTL −log10(P)
FMD GWAS −log10(P)
c
PP.abf = 85.8%
rs34573394
SMARCD1 − Artery − Aorta − eQTL −log10(P)
FMD GWAS −log10(P)
d
PP.abf =0%
LIMA1 − Artery − Tibial − eQTL −log10(P)
FMD GWAS −log10(P)

Figure S3. rs9349379-PHACTR1 eQTL in primary fibroblasts

Boxplot representation of *PHACTR1* normalized expression with respect to rs9349379 genotype (AA: red, AG: green, GG: blue) in primary fibroblasts from FMD patients. eQTL *P*-value is indicated. Middle line (bold) represents the median of data, box lower and upper limits represent the 25th and 75th centiles, respectively, and lower/upper whiskers represent smallest/largest observations greater than or equal to lower/upper hinge -/+ 1.5 times inter-quartile range.

Figure S3

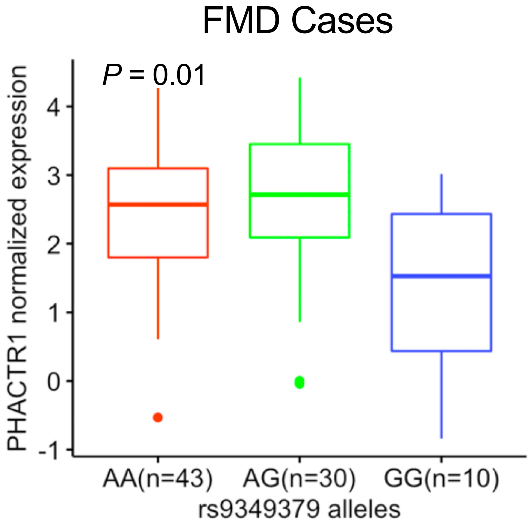

FMD Cases
P = 0.01
PHACTR1 normalized expression
4
3
2
1
0
-1
AA(n=43)
AG(n=30)
GG(n=10)
rs9349379 alleles

Figure S4. FMD association and eQTLs at *SLC24A3* locus

a-b: LocusZoom representation of FMD association signal at proximity of *SLC24A3* locus. Dot color indicates linkage disequilibrium of each variant with the highlighted lead variant (purple diamond). **a**: rs6046121. **b**: rs2424245. **c-e**: Violin plots representing normalized expression of *SLC24A3* in artery tissue by genotype of variant. **c**: rs6046121 in tibial artery. **d**: rs6046121 in aorta. **e**: rs2424245 in tibial artery. eQTL *P*-value is indicated. *: risk allele

Figure S4

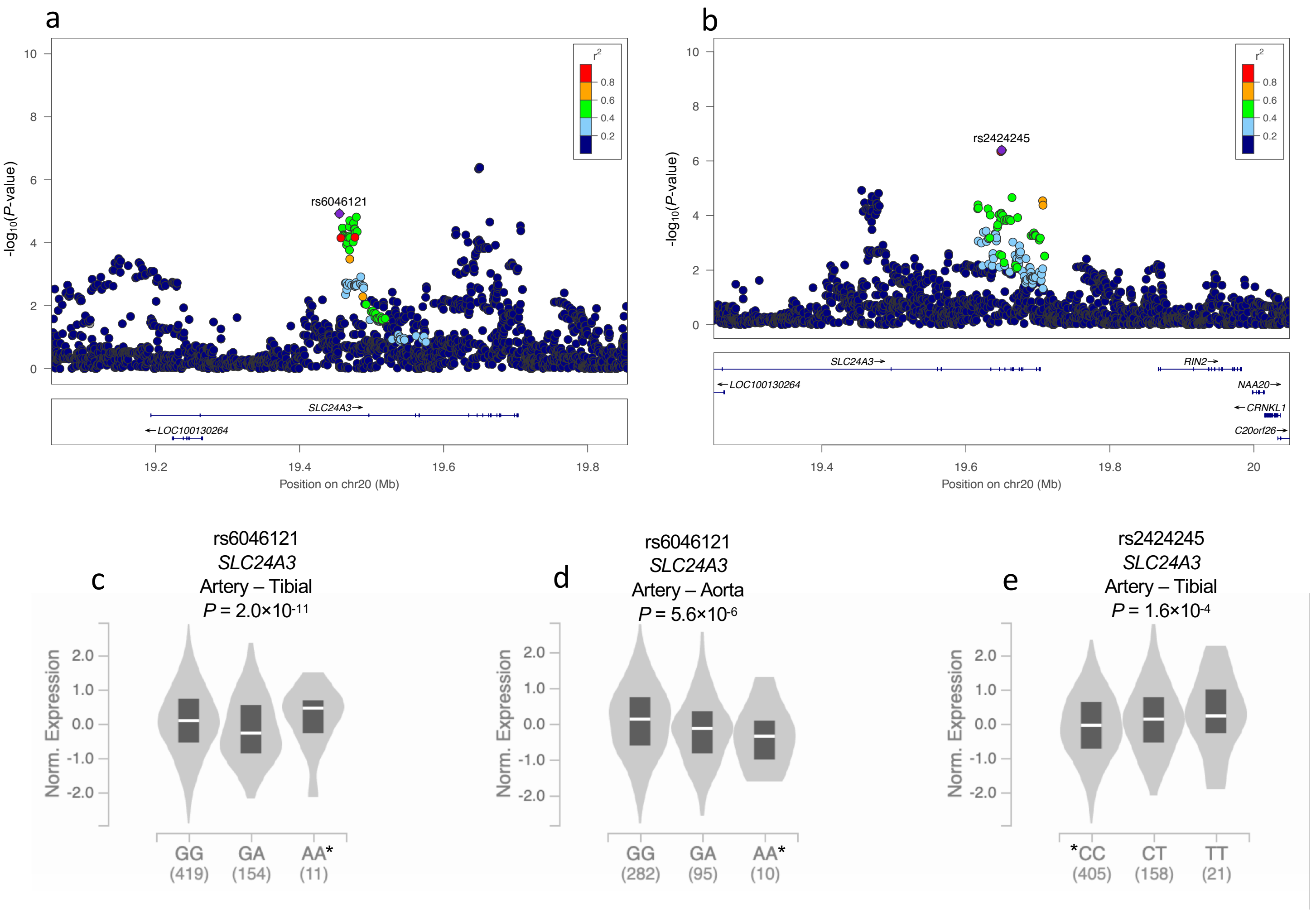

a
b
rs6046121
rs2424245
-log10(P-value)
r2
SLC24A3
LOC100130264
RIN2
NAA20
CRNKL1
C20orf26
Position on chr20 (Mb)
c
rs6046121
SLC24A3
Artery – Tibial
P = 2.0×10-11
GG (419)
GA (154)
AA* (11)
d
rs6046121
SLC24A3
Artery – Aorta
P = 5.6×10-6
GG (282)
GA (95)
AA* (10)
e
rs2424245
SLC24A3
Artery – Tibial
P = 1.6×10-4
*CC (405)
CT (158)
TT (21)
Norm. Expression

Figure S5. Characterization of open chromatin regions in artery derived primary cells

a: Number of reads (grey) and number of peaks (red) obtained for ATAC-Seq libraries from primary cells and artery tissue. HCtASMC: human carotid artery smooth muscle cells, HCASMC: human coronary artery smooth muscle cells, HCtAEC: human carotid artery endothelial cells, HCAEC: human coronary artery endothelial cells, HDF: human dermal fibroblasts, HCF: human cardiac fibroblasts, NCA: normal coronary arteries. NCA ATAC-Seq libraries were generated and sequenced by Miller and colleagues[1]. **b:** Heatmap representation of Spearman correlation and hierarchical clustering of ATAC-Seq datasets. The three main clusters correspond to VSMCs/fibroblasts, ECs and arteries, and are identified on the dendrogram. Rho correlation coefficient is represented by a red-blue colour scale and indicated in each box. **c:** Principal component analysis of ATAC-Seq datasets. Upper panel shows the position of samples with respect to first two principal components. Dot colour indicates the sample group: SMCs (orange), ECs (purple), fibroblasts (blue), artery tissue (grey). Dot shape indicates specific sample and is defined on the graph. Lower panel indicates the eigenvalues of the first 10 principal components. **d:** Representation of FMD SNPs fold-enrichment (x-axis) and enrichment *P*-value (log scale, y-axis) among indicated ATAC-Seq samples. The overlap of FMD lead SNPs ($P < 10^{-4}$ for the lead SNP) and proxies ($r^2 \geq 0.7$) with ATAC-Seq peaks was compared to 500 pools of randomized matched SNPs to calculate the indicated enrichments. Dot colour indicates the sample group: SMCs (orange), ECs (purple), fibroblasts (blue), artery tissue (grey). Dot shape indicates raw samples (square) or 50,000 top peaks from each groupe of samples (triangle). Lower panel shows the average fold enrichment in each group of samples. Error bars represent the standard deviation of 3 biologically independent samples. **e:** Bubble graph representing the clustering of enriched ($P < 10^{-3}$) gene ontology (GO) Biological Processes terms among genes (N=1425) located in the vicinity of artery-specific open chromatin regions (regions enriched over VSMCs and ECs). Similar terms were grouped

using REVIGO webserver (http://revigo.irb.hr/) with "Medium" setting. Bubble size indicates the number of enriched GO terms in each group. Bubble colour indicates the enrichment *P*-value of the most enriched term in each group. X- and Y-axes represent arbitrary semantic coordinates. **f**: Bar plot representing the enrichment score of the top 5 clusters obtained by Functional Annotation Clustering of the indicated 1425 genes using DAVID webserver (https://david.ncifcrf.gov/home.jsp). Most enriched term is indicated for each cluster.

Figure S5

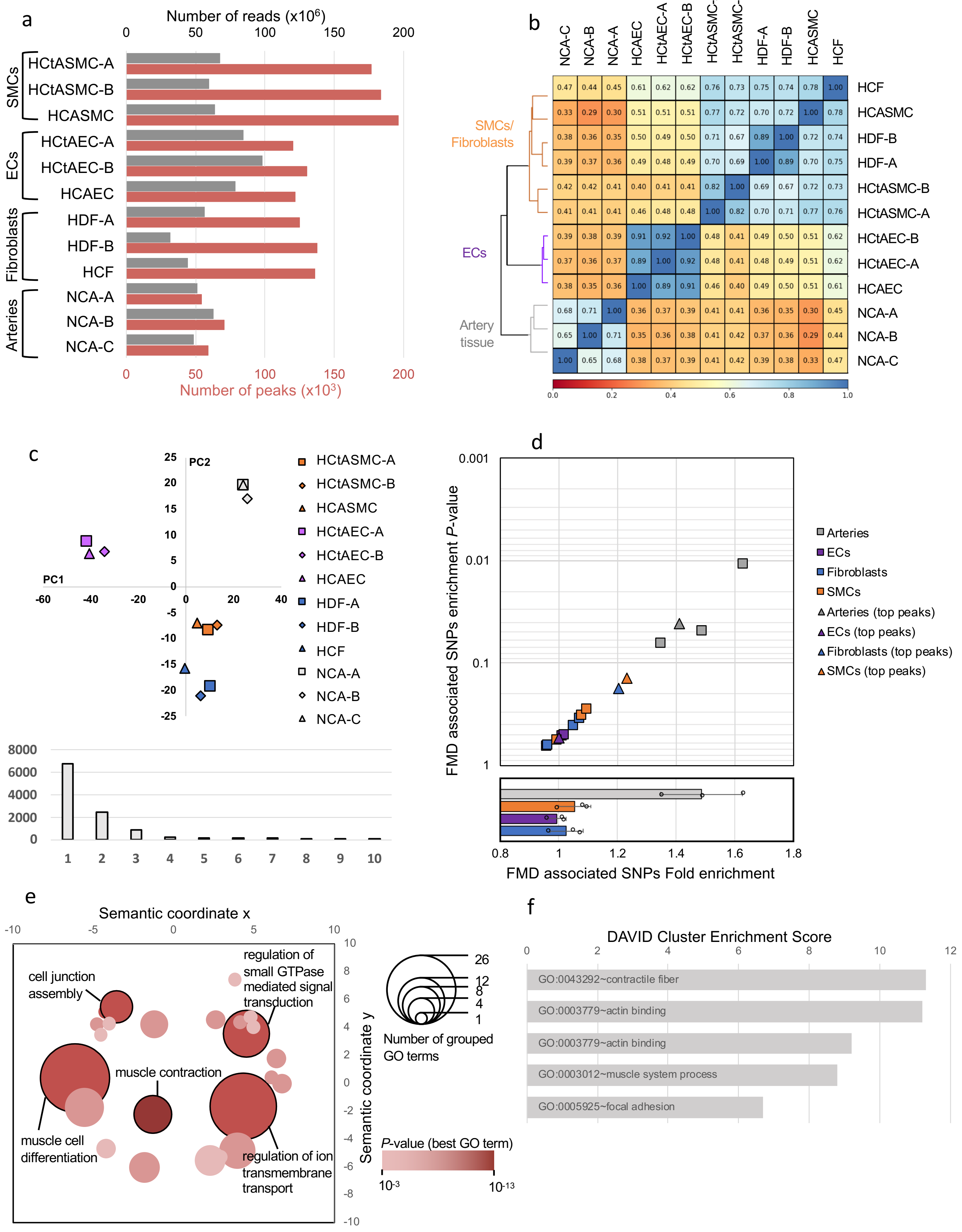
a
Number of reads (x10^6)
0 50 100 150 200
SMCs
HCtASMC-A
HCtASMC-B
HCASMC
ECs
HCtAEC-A
HCtAEC-B
HCAEC
Fibroblasts
HDF-A
HDF-B
HCF
Arteries
NCA-A
NCA-B
NCA-C
0 50 100 150 200
Number of peaks (x10^3)
b
NCA-C NCA-B NCA-A HCAEC HCtAEC-A HCtAEC-B HCtASMC-A HCtASMC-B HDF-A HDF-B HCASMC HCF
SMCs/ Fibroblasts
ECs
Artery tissue
0.47 0.44 0.45 0.61 0.62 0.62 0.76 0.73 0.75 0.74 0.78 1.00 HCF
0.33 0.29 0.30 0.51 0.51 0.51 0.77 0.72 0.70 0.72 1.00 0.78 HCASMC
0.38 0.36 0.35 0.50 0.49 0.50 0.71 0.67 0.89 1.00 0.72 0.74 HDF-B
0.39 0.37 0.36 0.49 0.48 0.49 0.70 0.69 1.00 0.89 0.70 0.75 HDF-A
0.42 0.42 0.41 0.40 0.41 0.41 0.82 1.00 0.69 0.67 0.72 0.73 HCtASMC-B
0.41 0.41 0.41 0.46 0.48 0.48 1.00 0.82 0.70 0.71 0.77 0.76 HCtASMC-A
0.39 0.38 0.39 0.91 0.92 1.00 0.48 0.41 0.49 0.50 0.51 0.62 HCtAEC-B
0.37 0.36 0.37 0.89 1.00 0.92 0.48 0.41 0.48 0.49 0.51 0.62 HCtAEC-A
0.38 0.35 0.36 1.00 0.89 0.91 0.46 0.40 0.49 0.50 0.51 0.61 HCAEC
0.68 0.71 1.00 0.36 0.37 0.39 0.41 0.41 0.36 0.35 0.30 0.45 NCA-A
0.65 1.00 0.71 0.35 0.36 0.38 0.41 0.42 0.37 0.36 0.29 0.44 NCA-B
1.00 0.65 0.68 0.38 0.37 0.39 0.41 0.42 0.39 0.38 0.33 0.47 NCA-C
0.0 0.2 0.4 0.6 0.8 1.0
c
PC2
PC1
25 20 15 10 5 0 -5 -10 -15 -20 -25
-60 -40 -20 0 20 40
HCtASMC-A
HCtASMC-B
HCASMC
HCtAEC-A
HCtAEC-B
HCAEC
HDF-A
HDF-B
HCF
NCA-A
NCA-B
NCA-C
8000 6000 4000 2000 0
1 2 3 4 5 6 7 8 9 10
d
FMD associated SNPs enrichment P-value
0.001 0.01 0.1 1
Arteries
ECs
Fibroblasts
SMCs
Arteries (top peaks)
ECs (top peaks)
Fibroblasts (top peaks)
SMCs (top peaks)
0.8 1 1.2 1.4 1.6 1.8
FMD associated SNPs Fold enrichment
e
Semantic coordinate x
-10 -5 0 5 10
Semantic coordinate y
10 8 6 4 2 0 -2 -4 -6 -8 -10
cell junction assembly
regulation of small GTPase mediated signal transduction
muscle contraction
muscle cell differentiation
regulation of ion transmembrane transport
26 12 8 4 1
Number of grouped GO terms
P-value (best GO term)
10-3 10-13
f
DAVID Cluster Enrichment Score
0 2 4 6 8 10 12
GO:0043292~contractile fiber
GO:0003779~actin binding
GO:0003779~actin binding
GO:0003012~muscle system process
GO:0005925~focal adhesion

Figure S6. Single-cell expression of FMD associated genes in mouse aorta

Violin plot representation of the expression of potential FMD associated genes in the single cell analysis of mouse aorta tissue by Kalluri and colleagues[2]. Unsupervised graph clustering was used to generate cell clusters, which were attributed to cell types according to the expression of canonical markers such as SMC markers *Myh11*, *Acta2 or Tagln.* Single-cell data were accessed and graphs were generated through Broad institute single cell portal (https://singlecell.broadinstitute.org/single_cell). Queried genes are indicated over each graph. Colour indicates cell subset and is defined on the graph. Query for *Gpd1* retrieved no results.

Figure S6

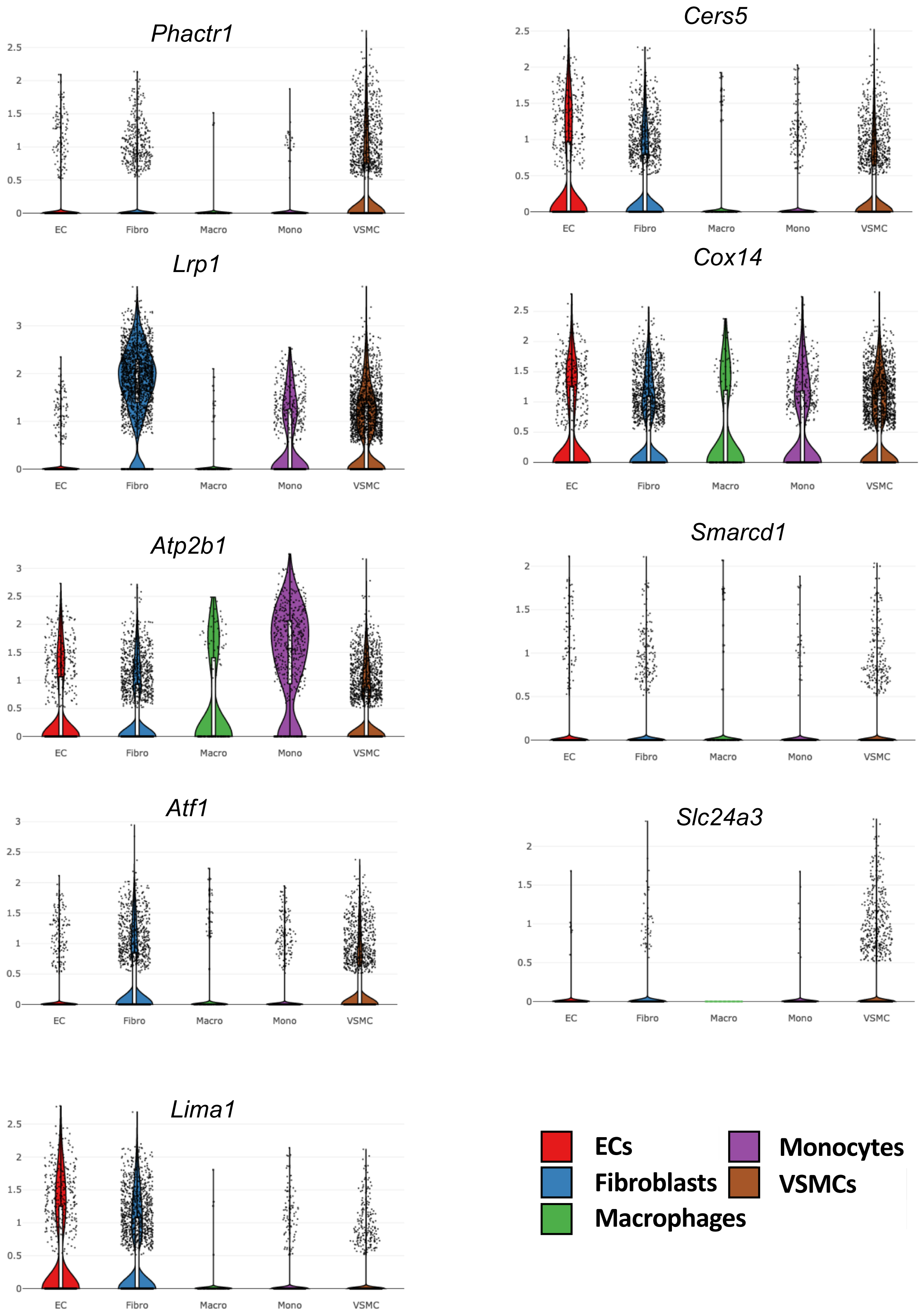

Phactr1
Cers5
Lrp1
Cox14
Atp2b1
Smarcd1
Atf1
Slc24a3
Lima1
EC
Fibro
Macro
Mono
VSMC
ECs
Fibroblasts
Macrophages
Monocytes
VSMCs

Figure S7. Gene expression of FMD associated genes in tibial artery tissue.

a: Box-plot representation of the expression (in tags-per-million, y-axis, log scale) of FMD associated genes (x-axis) in the 663 tibial artery samples from GTEx database. Middle line (bold) represents the median of data, box lower and upper limits represent the 25th and 75th centiles, respectively, and lower/upper whiskers represent smallest/largest observations greater than or equal to lower/upper hinge -/+ 1.5 times inter-quartile range. Colour indicates different genes genes and is defined on the graph. **b:** Violin-plot representation of the expression (in tags-per-million, y-axis, log scale) of FMD associated genes (x-axis) in the female (red, F, N = 209) and male (blue, M, N = 454) tibial artery samples from GTEx database. Student's two-sided t-test *P*-value is indicated on the graph.

Figure S7

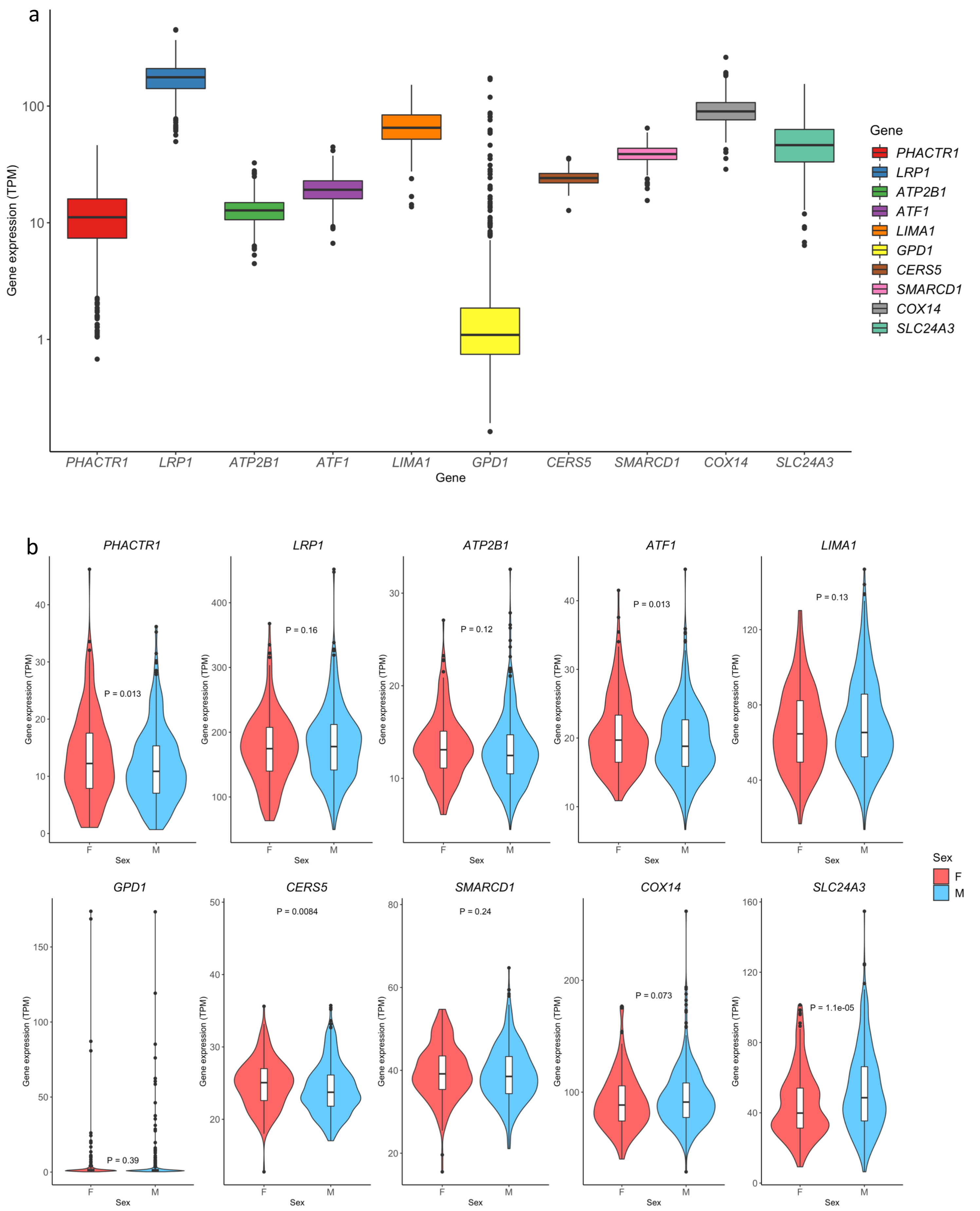

a
Gene expression (TPM)
100
10
1
PHACTR1
LRP1
ATP2B1
ATF1
LIMA1
GPD1
CERS5
SMARCD1
COX14
SLC24A3
Gene
b
P = 0.013
P = 0.16
P = 0.12
P = 0.013
P = 0.13
P = 0.39
P = 0.0084
P = 0.24
P = 0.073
P = 1.1e-05
Sex
F
M

SUPPLEMENTARY TABLES

Supplementary Table S1: Clinical characteristics of the study populations

BMI: Body-Mass Index, HTN: Hypertension, n: count, min: minimum range, max: maximum range, SD: Standard Deviation, y: years, MultiF: Multifocal, MultiS: Multisite, NA: not available, NR: not relevant

Country	Study	Type [status]	Inclusion criteria	Exclusion criteria	Total	Women	Age at inclusion (y)	Age of FMD diagnosis (y)	BMI (kg/m2)	HTN	Clinical presentation	FMD location MultiS/ Unisite/ NA
					n	n (%)	Mean ± SD [min -max]	Mean ± SD [min -max]	Mean ± SD	n (%)	Cerebral / Renal /other	n
France	ARCADIA	Clinical based [Cases]	Imaging-based FMD diagnosis	Heritable vascular diseases	431	376 (87.2)	55.68± 12.49 [19 - 89]	53.50 ± 13.09 [17 - 89]	23.74 ± 4.28	344 (79.8)	181/362/NA	162/265/4
France	3 Cities study	Population based [Controls]	Geographic sampling	Age < 65y	1487	876 (58.9)	74.36 ± 5.5 [65 - 94]	NR	NA	1171 (78.7)	NR	NR
Poland	ARCADIA-POL	Clinical based [Cases]	Imaging-based FMD diagnosis	Heritable vascular diseases	107	88 (82.2)	47.82 ± 13.23 [18 - 75]	45.35 ± 13.46 [18 - 75]	24.80 ± 4.01	91 (85%)	32/ 88/ 19	40/67/0
Poland	WOBASZ II	Population based [Controls]	Geographic sampling	None	295	227 (76.9)	40.97 ± 11.05 [21 - 86]	NR	26.37 ± 5.43	59	NR	NR
Europe	FEIRI	Clinical based [Cases]	Imaging-based FMD diagnosis	Primary spontaneous coronary artery dissection, heritable connective tissue disorders	243	215 (88.5)	NA	50.60 ± 12.96 [10 - 84]	NA	NA	129/175/71	108/132/3
Europe	ASKLEPIOS	Population based [Controls]	Age 35-55, Geographic sampling	Atherosclerosis, diabetes, other major illness	615	611 (99.3)	45.67 ± 6.23 [15 - 58]	NR	NA	NA	NR	NR
USA	MayoVDB	Clinical based [Cases]	Imaging-based FMD diagnosis	Heritable connective tissue disorders	116	100 (86.2)	58.78 ± 12.71 [27 - 85]	NA	26.87 ± 6.06	82 (70.7)	NA	NA
USA	MayoVDB	Clinical based [Controls]	18 years or older, able to consent	known FMD or atherosclerotic vascular diseases	1141	572 (50.1)	64.98 ± 11.30 [18 - 94]	NR	29.33 ± 6.02	680 (59.6)	NR	NR
USA	DEFINE-FMD	Clinical based [Cases]	Imaging-based FMD diagnosis	male sex, heritable vascular disease, major diseases, use of immunosuppressive agents, major comorbidities	108	108 (100.0)	56.13 ± 10.00 [32 - 80]	52.35 ± 10.49 [24 - 73]	24.13 ± 4.07	62 (51.4)	14/74/136	73 / 35 / 0
USA	DEFINE-FMD	Clinical based [Controls]	Matching by age & sex	≥2 blood pressure medications, BMI>35kg/m2, male sex, heritable vascular disease, major diseases, use of immunosuppressive agents, major comorbidities	126	126 (100.0)	49.49 ± 11.53 [23 - 74]	NR	23.99 + 3.86	NA	NR	NR
USA	UM/CCF	Clinical based [Cases]	Imaging-based FMD diagnosis	NR	551	534 (96.9)	53.77 ± 11.48 [11 - 84]	50.29 ± 11.54 [1 - 78]	25.13 ± 5.03	288 (52.2)	60/334/190	296/ 243 / 12
USA	UM-MGI	Population based [Controls]	Biobank (Matched by age, sex, smallest PC distances of PC1-PC3)	Vascular diseases and connective tissue disorders	3436	3312(96.4)	54.42 ± 12.46 [18 - 89]	NR	30.57 ± 9.23	1512 (44.0)	NR	NR

Supplementary table S2: Association of the top SNPs (P-Value < 5e-8) in chromosomes 6 and 12 with multifocal FMD

The table shows odds ratio, p-values and heterogeneity p-values of meta-analysis using Metal and results by studies.

CHROM: chromosome,POS: position,EA: effect allele, OA: other allele, EAF: effect allele frequency, OR: odds ratio(effect size), 95%CI: 95% confidence interval, P: p-value, Direction: effect direction in each study (FR,Mayo, Define, POL, UM and FEIRI), Het. *P* : heterogeneity *P* -value between studies.

Variant							Studies																			
							FR		Mayo		Define		POL		UM/CCF		FEIRI		meta-analysis				meta-analysis (women only)			
**CHROM**	**POS**	**rsID**	**Gene**	**EA**	**OA**	**EAF**	**OR**	**P**	**OR**	**P**	**OR**	**P**	**OR**	**P**	**OR**	**P**	**OR**	**P**	**OR (95%CI)**	**P**	**Direction**	**Het. P**	**OR (95%CI)**	**P**	**Direction**	**Het. P**
6	12903957	rs9349379	*PHACTR1*	A	G	0.62	1.55	9.1E-07	1.32	6.4E-02	1.37	1.2E-01	1.33	1.0E-01	1.43	2.7E-07	1.45	4.0E-02	1.44 (1.31 - 1.57)	5.2E-15	++++++	0.93	1.45 (1.32 - 1.60)	1.8E-14	++++++	0.76
12	57527283	rs11172113	*LRP1*	T	C	0.62	1.22	2.6E-02	1.62	1.8E-03	1.30	2.0E-01	1.07	6.8E-01	1.40	7.5E-07	1.46	2.4E-02	1.34 (1.22 - 1.46)	2.0E-10	++++++	0.39	1.32 (1.21 - 1.45)	5.2E-09	++++++	0.39
12	50581647	rs7301566	*LIMA1*	T	C	0.45	1.16	6.7E-02	1.32	5.7E-02	1.60	1.8E-02	1.52	1.7E-02	1.35	3.2E-06	1.09	6.1E-01	1.29 (1.19 - 1.41)	3.8E-09	++++++	0.39	1.32 (1.21 - 1.45)	1.3E-09	++++++	0.32
12	50593859	rs2004283	*LIMA1*	T	G	0.45	1.14	1.1E-01	1.35	4.3E-02	1.64	1.3E-02	1.46	2.9E-02	1.35	2.8E-06	1.07	6.6E-01	1.29 (1.18 - 1.40)	7.7E-09	++++++	0.30	1.31 (1.20 - 1.43)	4.0E-09	++++++	0.28
12	50594947	rs4459386	*LIMA1(NM_001243775: c.-322T>C)*	A	G	0.44	1.15	8.3E-02	1.35	4.3E-02	1.64	1.3E-02	1.46	2.9E-02	1.35	2.9E-06	1.08	6.5E-01	1.29 (1.18 - 1.40)	5.2E-09	++++++	0.33	1.31 (1.20 - 1.44)	2.9E-09	++++++	0.30
12	50595445	rs10219559	*LIMA1*	T	C	0.44	1.14	1.1E-01	1.35	4.3E-02	1.64	1.3E-02	1.46	2.9E-02	1.35	2.9E-06	1.08	6.5E-01	1.29 (1.18 - 1.40)	7.5E-09	++++++	0.31	1.31 (1.20 - 1.43)	4.1E-09	++++++	0.28
12	50611020	rs8181679	*LIMA1*	T	C	0.44	1.15	8.3E-02	1.35	4.2E-02	1.64	1.3E-02	1.45	3.1E-02	1.35	3.4E-06	1.06	7.2E-01	1.29 (1.18 - 1.40)	6.9E-09	++++++	0.32	1.31 (1.20 - 1.43)	3.8E-09	++++++	0.29
12	50628466	rs10783342	*LIMA1*	T	C	0.45	1.12	1.5E-01	1.35	4.2E-02	1.64	1.3E-02	1.45	3.1E-02	1.36	2.6E-06	1.06	7.2E-01	1.28 (1.18 - 1.40)	1.4E-08	++++++	0.24	1.30 (1.19 - 1.43)	6.8E-09	++++++	0.23
12	90008959	rs2681472	*ATP2B1*	A	G	0.84	1.42	2.5E-03	1.23	3.2E-01	1.37	2.7E-01	1.28	3.2E-01	1.49	3.7E-05	1.45	8.9E-02	1.42 (1.26 - 1.61)	2.7E-08	++++++	0.97	1.45 (1.27 - 1.65)	3.1E-08	++++++	0.92
12	90013089	rs2681492	*ATP2B1*	T	C	0.84	1.42	2.5E-03	1.23	3.2E-01	1.42	2.2E-01	1.29	3.2E-01	1.51	2.5E-05	1.45	9.1E-02	1.43 (1.26 - 1.62)	1.7E-08	++++++	0.96	1.46 (1.28 - 1.66)	1.9E-08	++++++	0.91
12	90026462	rs11105352	*ATP2B1*	G	A	0.84	1.46	1.7E-03	1.22	3.3E-01	1.37	2.7E-01	1.29	3.2E-01	1.48	4.7E-05	1.45	9.3E-02	1.43 (1.26 - 1.62)	2.6E-08	++++++	0.97	1.45 (1.27 - 1.65)	3.9E-08	++++++	0.91
12	90026463	rs11105353	*ATP2B1*	C	A	0.84	1.46	1.7E-03	1.22	3.3E-01	1.37	2.7E-01	1.29	3.2E-01	1.48	4.7E-05	1.45	9.3E-02	1.43 (1.26 - 1.62)	2.6E-08	++++++	0.97	1.45 (1.27 - 1.65)	3.9E-08	++++++	0.91
12	90026523	rs11105354	*ATP2B1*	A	G	0.84	1.43	2.3E-03	1.23	3.1E-01	1.37	2.7E-01	1.29	3.2E-01	1.50	3.1E-05	1.45	9.0E-02	1.43 (1.26 - 1.62)	2.1E-08	++++++	0.96	1.45 (1.27 - 1.65)	3.2E-08	++++++	0.92
12	90050503	rs12579302	*ATP2B1*	A	G	0.84	1.42	3.3E-03	1.22	3.3E-01	1.37	2.7E-01	1.29	3.2E-01	1.50	2.8E-05	1.45	9.1E-02	1.42 (1.26 - 1.61)	2.9E-08	++++++	0.96	1.44 (1.27 - 1.65)	4.3E-08	++++++	0.91
12	90054619	rs73437338	*ATP2B1(dist=4775), LINC00936(dist=48113)*	T	C	0.84	1.40	4.9E-03	1.24	3.0E-01	1.38	2.6E-01	1.25	3.8E-01	1.55	1.0E-05	1.46	8.8E-02	1.44 (1.27 - 1.63)	1.9E-08	++++++	0.92	1.46 (1.28 - 1.67)	2.9E-08	++++++	0.91
12	90058842	rs111478946	*ATP2B1(dist=8998), LINC00936(dist=43890)*	G	A	0.84	1.41	3.3E-03	1.22	3.3E-01	1.42	2.2E-01	1.29	3.2E-01	1.50	2.8E-05	1.45	9.0E-02	1.43 (1.26 - 1.61)	2.5E-08	++++++	0.96	1.45 (1.27 - 1.65)	3.6E-08	++++++	0.91
12	90060586	rs17249754	*ATP2B1(dist=10742), LINC00936(dist=42146)*	G	A	0.84	1.41	3.3E-03	1.23	3.1E-01	1.47	1.8E-01	1.28	3.2E-01	1.51	2.3E-05	1.45	8.8E-02	1.43 (1.26 - 1.62)	1.7E-08	++++++	0.96	1.45 (1.27 - 1.66)	2.4E-08	++++++	0.91
12	90062376	rs6538195	*ATP2B1(dist=12532), LINC00936(dist=40356)*	G	A	0.84	1.41	3.3E-03	1.23	3.1E-01	1.47	1.8E-01	1.28	3.2E-01	1.51	2.3E-05	1.45	9.0E-02	1.43 (1.26 - 1.62)	1.8E-08	++++++	0.96	1.45 (1.27 - 1.66)	2.5E-08	++++++	0.91
12	90069276	rs11105364	*ATP2B1(dist=19432), LINC00936(dist=33456)*	T	G	0.84	1.41	3.6E-03	1.22	3.3E-01	1.42	2.2E-01	1.28	3.2E-01	1.50	2.8E-05	1.45	9.0E-02	1.42 (1.26 - 1.61)	2.9E-08	++++++	0.96	1.44 (1.27 - 1.65)	4.0E-08	++++++	0.91
12	90072076	rs73437358	*ATP2B1(dist=22232), LINC00936(dist=30656)*	G	A	0.84	1.39	5.1E-03	1.22	3.3E-01	1.47	1.8E-01	1.27	3.4E-01	1.51	2.3E-05	1.46	8.9E-02	1.42 (1.26 - 1.61)	3.0E-08	++++++	0.94	1.44 (1.27 - 1.65)	4.2E-08	++++++	0.89
12	90074441	rs11105368	*ATP2B1(dist=24597), LINC00936(dist=28291)*	G	C	0.84	1.39	5.1E-03	1.22	3.3E-01	1.47	1.8E-01	1.27	3.4E-01	1.50	2.7E-05	1.46	8.9E-02	1.42 (1.25 - 1.61)	3.3E-08	++++++	0.95	1.44 (1.26 - 1.65)	4.8E-08	++++++	0.90
12	90075243	rs4842675	*ATP2B1(dist=25399), LINC00936(dist=27489)*	C	T	0.84	1.39	5.1E-03	1.22	3.3E-01	1.47	1.8E-01	1.27	3.4E-01	1.50	2.8E-05	1.46	8.9E-02	1.42 (1.25 - 1.61)	3.5E-08	++++++	0.95	1.44 (1.26 - 1.64)	5.0E-08	++++++	0.90

Supplementary Table S3: Gene-based associated genes with FMD

Genes with gene-based association FDR < 0.05 are shown. Chr: chromosome, P.Bonf : Bonferroni corrected P-value. FDR: False Discovery Rate

Gene	Chr	Start	End	P	P.bonf	FDR
ATF1	12	51157493	51214905	4.0E-07	0.01	0.003
ATP2B1	12	89981828	90103077	4.8E-07	0.01	0.003
GPD1	12	50497602	50505102	5.0E-07	0.01	0.003
CERS5	12	50523575	50561288	1.4E-06	0.03	0.01
LIMA1	12	50569571	50677329	2.3E-06	0.04	0.01
SUSD1	9	114803065	114937688	1.3E-05	0.23	0.04
ZNF827	4	146678779	146859787	1.5E-05	0.27	0.04
FHL5	6	97010424	97064512	1.7E-05	0.31	0.04

Supplementary Table S4: Colocalization of association with FMD and eQTL association.

We display the results of approxiate Bayes factor colocalization analysis for the association with FMD, on one hand, eQTL association with the indicated eGenes, on the other hand.

NSNPs: number of SNPs used for he analysis. All SNPs present in both studies in a 2Mb window centered on FMD lead variant were used. H0-H4: Posterior probability that: H0: neither trait has a genetic association in the region; H1: only trait 1 has a genetic association in the region; H2: only trait 2 has a genetic association in the region; H3: both traits are associated, but with different causal variants; H4: both traits are associated and share a single causal variant.

mFMD lead SNP	eGene	Tissue	mFMD lead SNP eQTL P-value	Best eQTL SNP	Best eQTL P-value	NSNPs	H0	H1	H2	H3	H4
rs9349379	PHACTR1	Artery - Tibial	8.0E-42	rs9349379	8.0E-42	3294	0.0%	0.0%	0.0%	0.0%	100.0%
rs9349379	PHACTR1	Artery - Aorta	2.0E-17	rs9349379	2.0E-17	3294	0.0%	0.0%	0.0%	0.0%	100.0%
rs9349379	PHACTR1	Artery - Coronary	3.0E-09	rs9349379	3.0E-09	3294	0.0%	0.0%	0.0%	0.0%	100.0%
rs9349379	TBC1D7	Artery - Tibial	1.6E-06	rs499818	2.1E-11	2809	0.0%	0.0%	0.0%	98.2%	1.8%
rs9349379	GFOD1	Artery - Tibial	1.1E-04	rs9357720	3.9E-16	2491	0.0%	0.0%	0.0%	100.0%	0.0%
rs11172113	LRP1	Artery - Tibial	9.4E-21	rs11172113	9.4E-21	1691	0.0%	0.0%	0.0%	0.0%	100.0%
rs11172113	LRP1	Artery - Aorta	3.6E-15	rs11172113	3.6E-15	1691	0.0%	0.0%	0.0%	0.0%	100.0%
rs2681492	ATP2B1	Artery - Tibial	3.4E-18	rs2681472	1.6E-18	2514	0.0%	0.0%	0.0%	1.7%	98.3%
rs2681492	ATP2B1-AS1	Artery - Tibial	1.2E-05	rs11105328	2.0E-06	2514	0.0%	0.7%	0.0%	2.7%	96.6%
rs2681492	ATP2B1	Artery - Aorta	6.6E-05	rs7302816	1.6E-05	2514	0.0%	4.4%	0.0%	3.5%	92.0%
rs7301566	ATF1	Artery - Aorta	6.9E-14	rs11169571	5.2E-36	1523	0.0%	0.0%	0.1%	94.1%	5.8%
rs7301566	ATF1	Artery - Tibial	6.2E-12	rs11169571	3.1E-48	1523	0.0%	0.0%	0.1%	90.4%	9.5%
rs7301566	COX14	Artery - Aorta	3.3E-06	rs10783340	9.6E-13	2187	0.0%	0.0%	0.1%	99.8%	0.1%
rs7301566	SMARCD1	Artery - Aorta	2.7E-05	rs34573394	7.9E-07	2141	0.0%	0.6%	0.0%	13.5%	85.8%
rs7301566	LIMA1	Artery - Tibial	9.1E-05	rs17124706	3.3E-21	2192	0.0%	0.0%	0.1%	99.9%	0.0%
rs2424245	SLC24A3	Artery - Tibial	1.6E-04	rs3790227	2.1E-22	3558	0.0%	0.0%	8.7%	19.4%	71.9%
rs6046121	SLC24A3	Artery - Tibial	2.0E-11	rs3790227	2.1E-22	4015	0.0%	0.0%	8.7%	19.6%	71.7%
rs6046121	SLC24A3	Artery - Aorta	5.6E-06	rs1984571	2.5E-12	4015	0.0%	0.0%	12.0%	27.1%	60.8%

Supplementary Table S5: Transcriptome-wide associated genes with FMD in 3 artery tissues (GTEx v7 gene expression models).
Genes with TWAS FDR < 0.05 are shown. Chr: chromosome, P.Bonf : Bonferroni corrected P-value. FDR: False Discovery Rate

Gene	Tissue	Chr	Start	End	Best GWAS SNP	Best GWAS SNP Z-Score	Best eQTL SNP	Best eQTL SNP Z-Score	TWAS Z-score	TWAS P-value	TWAS P.Bonf	TWAS FDR
PHACTR1	Artery_Aorta	6	12717893	13288645	rs9349379	-7.83	rs9349379	-6.21	7.31	2.77E-13	3.9E-09	3.9E-09
PHACTR1	Artery_Tibial	6	12717893	13288645	rs9349379	-7.83	rs9349379	-9.18	7.20	5.95E-13	8.5E-09	4.2E-09
LRP1	Artery_Tibial	12	57522276	57607134	rs11172113	-6.36	rs11172113	-8.24	6.36	2.01E-10	2.9E-06	9.6E-07
SLC24A3	Artery_Tibial	20	19193290	19703581	rs2424245	-4.6	rs3790227	-5.92	-4.99	5.89E-07	0.008	0.002
LEFTY1	Artery_Aorta	1	226073982	226099082	rs4653694	4.64	rs4653694	-4.93	-4.61	4.08E-06	0.06	0.011
ARL17A	Artery_Coronary	17	44594068	44657088	rs199454	-4.93	rs17692129	-3.98	-4.58	4.66E-06	0.07	0.011
NSF	Artery_Coronary	17	44668035	44834830	rs199454	-4.93	rs17698176	3.94	4.43	9.53E-06	0.14	0.019
ATP2B1	Artery_Tibial	12	89981828	90102608	rs2681492	-5.64	rs2681492	7.12	-4.36	1.29E-05	0.18	0.023
FGFR2	Artery_Tibial	10	123237848	123357972	rs7072877	4.76	rs10788184	7.78	4.31	1.63E-05	0.23	0.026
MMAA	Artery_Aorta	4	146539415	146581187	rs11100901	4.55	rs9790518	3.61	4.26	2.07E-05	0.30	0.026
NSF	Artery_Aorta	17	44668035	44834830	rs199454	-4.93	rs35732828	3.68	4.24	2.27E-05	0.32	0.026
LRRC37A2	Artery_Coronary	17	44588877	44630815	rs199454	-4.93	rs199451	7.96	-4.23	2.37E-05	0.34	0.026
ATF1	Artery_Aorta	12	51157493	51214905	rs7967954	5.92	rs7306677	8.27	-4.22	2.42E-05	0.35	0.026
LRRC37A2	Artery_Aorta	17	44588877	44630815	rs199454	-4.93	rs199456	10.99	-4.21	2.53E-05	0.36	0.026
FGFR2	Artery_Aorta	10	123237848	123357972	rs7072877	4.76	rs10788184	6.95	4.16	3.16E-05	0.45	0.030
DMWD	Artery_Coronary	19	46286205	46296060	rs8103278	-4.04	rs10406475	3.78	4.07	4.69E-05	0.67	0.042
GUCY1A3	Artery_Aorta	4	156587863	156653501	rs17033041	4.2	rs17033041	-7.51	-4.03	5.64E-05	0.80	0.045
ZNF827	Artery_Tibial	4	146678779	146859787	rs11100901	4.55	rs1979974	7.49	4.03	5.68E-05	0.81	0.045
ARL17A	Artery_Aorta	17	44594068	44657088	rs199454	-4.93	rs199456	4.79	-3.99	6.64E-05	0.95	0.050

Supplementary Table S6: Association of FMD loci unadjusted, adjusted for and stratified by hypertension status.
We display odd ratios (ORs) and P-values estimates for the association of the lead SNPs obtained the French and UM/CCF studies for the main GWAS loci. HTN: hypertension.

						French study					UM/CCF study				
	**Locus**	**Lead SNP**	**Chr**	**Pos**	**Effect allele**	**Total**	**Cases**	**Controls**	**ORs**	**P-value**	**Total**	**Cases**	**Controls**	**ORs**	**P-value**
**Original association**	*PHACTR1*	rs9349379	*6*	*1.3E+07*	A	1918	431	1487	1.55	9.1E-07	3987	551	3436	1.45	1.5E-07
	LIMA1	rs7301566	*12*	*5.1E+07*	T	1918	431	1487	1.16	0.07	3987	551	3436	1.37	2.0E-06
	LRP1	rs11172113	*12*	*5.8E+07*	T	1918	431	1487	1.22	0.03	3987	551	3436	1.40	1.2E-06
	ATP2B1	rs2681492	*12*	*9E+07*	T	1918	431	1487	1.42	2.5E-03	3987	551	3436	1.50	3.9E-05
**Association adjusted for HTN status**	*PHACTR1*	rs9349379	*6*	*1.3E+07*	A	1918	431	1485	1.55	1.2E-06	3639	551	3088	1.44	2.2E-07
	LIMA1	rs6580732	*12*	*5.1E+07*	T	1918	431	1485	1.16	0.07	3639	551	3088	1.36	5.0E-06
	LRP1	rs11172113	*12*	*5.8E+07*	T	1918	431	1485	1.23	0.02	3639	551	3088	1.39	2.2E-06
	ATP2B1	rs17249754	*12*	*9E+07*	T	1918	431	1485	1.42	2.5E-03	3639	551	3088	1.50	4.4E-05
**HTN cases and controls**	*PHACTR1*	rs9349379	*6*	*1.3E+07*	A	1519	348	1171	1.67	3.6E-07	1800	288	1512	1.27	1.3E-02
	LIMA1	rs6580732	*12*	*5.1E+07*	T	1519	348	1171	1.18	0.08	1800	288	1512	1.45	7.9E-05
	LRP1	rs11172113	*12*	*5.8E+07*	T	1519	348	1171	1.29	9.6E-03	1800	288	1512	1.38	8.1E-04
	ATP2B1	rs17249754	*12*	*9E+07*	T	1519	348	1171	1.47	3.5E-03	1800	288	1512	1.49	4.4E-03
**Non-HTN cases and controls**	*PHACTR1*	rs9349379	*6*	*1.3E+07*	A	397	83	314	1.17	0.44	1839	263	1576	1.67	1.0E-06
	LIMA1	rs6580732	*12*	*5.1E+07*	T	397	83	314	1.14	0.46	1839	263	1576	1.28	9.4E-03
	LRP1	rs11172113	*12*	*5.8E+07*	T	397	83	314	1.01	0.97	1839	263	1576	1.42	6.0E-04
	ATP2B1	rs17249754	*12*	*9E+07*	T	397	83	314	1.35	0.24	1839	263	1576	1.50	3.8E-03

Supplementary Table S7: Colocalization of association with FMD and five other traits at FMD top loci

We display the results of approxiate Bayes factor colocalization analysis for the association with FMD, on one hand, and five different traits (pulse pressure (PP), systolic blood pressure (SBP), coronry artey disease (CAD), migraine and cervical artery dissection (CeAD)), on the other hand.

NSNPs: number of SNPs used for he analysis. All SNPs present in both studies in a 2Mb window centered on FMD lead variant were used. H0-H4: Posterior probability that: H0: neither trait has a genetic association in the region; H1: only trait 1 has a genetic association in the region; H2: only trait 2 has a genetic association in the region; H3: both traits are associated, but with different causal variants; H4: both traits are associated and share a single causal variant.

mFMD leadSNP	Compared Trait	Best SNP fo compared trait	P-value of best SNP	NSNPs	H0	H1	H2	H3	H4
rs9349379	PP	rs9349379	1.3E-36	3721	0.0%	0.0%	0.0%	0.0%	100.0%
	SBP	rs9349379	1.3E-17	3721	0.0%	0.0%	0.0%	0.0%	100.0%
	CAD	rs9349379	1.8E-42	3727	0.0%	0.0%	0.0%	0.0%	100.0%
	Migraine	rs9349379	5.4E-15	3717	0.0%	0.0%	0.0%	0.0%	100.0%
	CeAD	rs9349379	1.2E-09	3449	0.0%	0.0%	0.0%	0.0%	100.0%
rs11172113	PP	rs11172113	3.0E-08	1746	0.0%	0.0%	0.0%	0.4%	99.6%
	SBP	rs2169987	1.2E-08	1743	0.0%	2.4%	0.0%	97.6%	0.0%
	CAD	rs11172113	1.7E-05	1726	0.0%	2.0%	0.0%	0.7%	97.3%
	Migraine	rs11172113	4.0E-24	1731	0.0%	0.0%	0.0%	0.0%	100.0%
	CeAD	rs11172113	5.1E-08	1598	0.0%	0.0%	0.0%	0.2%	99.8%
rs7301566	PP	rs117945311	4.8E-11	2359	0.0%	0.0%	0.1%	94.9%	5.1%
	SBP	rs12426261	2.3E-34	2359	0.0%	0.0%	0.0%	15.3%	84.7%
	CAD	rs9919739	6.4E-03	2357	0.1%	96.1%	0.0%	3.3%	0.5%
	Migraine	rs112643495	3.8E-03	2366	0.1%	99.7%	0.0%	0.2%	0.0%
	CeAD	rs4768862	8.3E-05	2108	0.0%	17.5%	0.0%	28.6%	53.9%
rs2681492	PP	rs111478946	8.2E-63	2580	0.0%	0.0%	0.0%	1.8%	98.2%
	SBP	rs17249754	1.3E-97	2580	0.0%	0.0%	0.0%	1.5%	98.5%
	CAD	rs2681472	6.2E-11	2579	0.0%	0.0%	0.0%	1.7%	98.3%
	Migraine	rs2165036	1.6E-03	2546	0.1%	99.4%	0.0%	0.4%	0.0%
	CeAD	rs2408160	1.8E-04	2343	0.1%	63.2%	0.1%	32.0%	4.7%
rs2424245	PP	rs6035355	1.2E-36	4162	0.0%	0.0%	15.7%	35.3%	49.0%
	SBP	rs3058639	1.7E-08	4163	0.2%	0.4%	14.8%	33.5%	51.0%
	CAD	rs73126920	3.6E-04	4131	26.1%	58.8%	3.8%	8.6%	2.7%
	Migraine	rs3827986	2.0E-08	4162	1.3%	2.9%	12.0%	27.1%	56.8%
	CeAD	rs6046289	1.8E-03	3755	24.3%	53.7%	6.2%	13.8%	1.9%

Supplementary Table S8: Genetic correlation between FMD and cardiovascular and neurovascular diseases and traits

rg: genetic correlation, se: standard error, P: P-value for the genetic correlation. eGFR: estimated glomerular filtration rate.

Disease or trait	rg	se	L95_rg	U95_rg	z	P
Hypertension	0.36	0.07	0.22	0.49	5.23	1.7E-07
Systolic Blood Pressure	0.43	0.07	0.29	0.57	5.99	2.1E-09
Diastolic Blood Pressure	0.37	0.07	0.25	0.50	5.72	1.1E-08
Pulse Pressure	0.35	0.06	0.23	0.48	5.60	2.1E-08
Coronary artery disease	-0.12	0.06	-0.23	0.00	-2.00	4.6E-02
Myocardial infarction	-0.12	0.07	-0.25	0.02	-1.71	8.8E-02
Migraine	0.28	0.08	0.12	0.44	3.34	8.4E-04
Any stroke	0.17	0.09	0.00	0.34	2.01	4.4E-02
Any ischemic stroke	0.19	0.08	0.03	0.35	2.27	2.3E-02
Large artery stroke	0.32	0.29	-0.24	0.88	1.14	2.6E-01
Cardioembolic stroke	0.38	0.12	0.14	0.63	3.12	1.8E-03
Small vessel stroke	0.22	0.16	-0.09	0.54	1.40	1.6E-01
Intracranial aneurysm	0.36	0.08	0.20	0.52	4.31	1.6E-05
Subarachnoid haemorrhage	0.35	0.10	0.17	0.54	3.69	2.2E-04
Unruptured intracranial aneurysm	0.33	0.12	0.10	0.57	2.78	5.4E-03
Cervical artery dissection	0.67	0.31	0.07	1.26	2.18	3.0E-02
High density lipopoteins	-0.03	0.04	-0.12	0.05	-0.75	4.5E-01
Low density lipoproteins	-0.18	0.06	-0.30	-0.06	-3.03	2.5E-03
Total cholesterol	-0.15	0.06	-0.26	-0.04	-2.66	7.8E-03
Tryglicerydes	0.09	0.05	0.00	0.18	2.01	4.4E-02
Apolipoprotein A	0.00	0.05	-0.09	0.09	-0.02	9.8E-01
Apolipoprotein B	-0.17	0.06	-0.28	-0.05	-2.82	4.7E-03
Creatinine	0.03	0.05	-0.06	0.13	0.71	4.8E-01
CystatinC	0.02	0.04	-0.06	0.09	0.43	6.7E-01
eGFR creatinine	0.02	0.05	-0.07	0.11	0.41	6.8E-01
eGFR cystatinC	0.06	0.09	-0.11	0.24	0.71	4.8E-01
Urine albumin to creatinin ratio	0.08	0.05	-0.02	0.19	1.54	1.2E-01
Blood urea nitrogen	0.02	0.06	-0.10	0.13	0.27	7.9E-01
Urate	0.01	0.04	-0.07	0.10	0.26	7.9E-01
Urea	0.04	0.05	-0.05	0.13	0.80	4.2E-01
C-reactive protein	-0.09	0.06	-0.20	0.03	-1.52	1.3E-01

Supplementary Table S9: Association of the lead SNPs in genome-wide associated loci with FMD before and after conditionning on SBP using GCTA-mtCOJO

BETA: effect size, SE: standard error of the effect size, P: P-value for the association. SBP: systolic blood pressure

						Original meta-analysis			SBP conditionned meta-analysis		
Locus	**rsID**	**Effect allele**	**Total**	**Cases**	**Controls**	**BETA**	**SE**	**P-value**	**BETA**	**SE**	**P-value**
PHACTR1	rs9349379	A	8678	1578	7100	0.36	0.05	5.2E-15	0.34	0.05	1.9E-13
LIMA1	rs7301566	T	8678	1578	7100	0.26	0.04	2.2E-09	0.23	0.04	1.6E-07
LRP1	rs11172113	T	8678	1578	7100	0.29	0.05	2.0E-10	0.30	0.05	1.2E-10
ATP2B1	rs2681492	T	8678	1578	7100	0.36	0.06	1.7E-08	0.29	0.06	4.5E-06

Supplementary Table S10: Genetic correlation between FMD and cardiovascular and neurovascular diseases and traits conditionned on systolic blood pressure genetic association data.
rg: genetic correlation, se: standard error, P: P-value for the genetic correlation. eGFR: estimated glomerular filtration rate.

**Disease or trait**	**rg**	**se**	**L95_rg**	**U95_rg**	**z**	**P**
Coronary artery disease	-0.31	0.08	-0.47	-0.16	-4.07	4.8E-05
Myocardial infarction	-0.30	0.08	-0.46	-0.13	-3.53	4.2E-04
Migraine	0.33	0.09	0.15	0.51	3.55	3.9E-04
Any stroke	-0.01	0.09	-0.19	0.16	-0.15	8.8E-01
Any ischemic stroke	0.01	0.08	-0.15	0.18	0.16	8.8E-01
Large artery stroke	0.01	0.25	-0.47	0.50	0.06	9.5E-01
Cardioembolic stroke	0.35	0.13	0.09	0.60	2.68	7.3E-03
Small vessel stroke	0.07	0.17	-0.27	0.40	0.39	7.0E-01
Intracranial aneurysm	0.27	0.09	0.10	0.45	3.06	2.2E-03
Subarachnoid haemorrhage	0.26	0.10	0.06	0.46	2.55	1.1E-02
Unruptured intracranial aneurysm	0.26	0.13	0.00	0.52	1.98	4.8E-02
Cervical artery dissection	0.60	0.34	-0.07	1.27	1.76	7.8E-02
High density lipopoteins	-0.03	0.05	-0.12	0.07	-0.57	5.7E-01
Low density lipoproteins	-0.13	0.06	-0.25	0.00	-1.99	4.7E-02
Total cholesterol	-0.11	0.06	-0.23	0.01	-1.80	7.2E-02
Tryglicerydes	0.06	0.05	-0.04	0.16	1.14	2.5E-01
Apolipoprotein A	-0.02	0.05	-0.12	0.08	-0.47	6.4E-01
Apolipoprotein B	-0.14	0.06	-0.27	-0.01	-2.18	3.0E-02
Creatinine	0.05	0.06	-0.06	0.15	0.83	4.1E-01
CystatinC	0.00	0.04	-0.08	0.09	0.05	9.6E-01
eGFR creatinine	0.03	0.05	-0.07	0.13	0.56	5.7E-01
eGFR cystatinC	0.11	0.10	-0.08	0.31	1.14	2.6E-01
Urine albumin to creatinin ratio	-0.09	0.05	-0.20	0.01	-1.74	8.2E-02
Blood urea nitrogen	-0.02	0.06	-0.13	0.10	-0.26	8.0E-01
Urate	-0.06	0.05	-0.16	0.04	-1.17	2.4E-01
Urea	0.01	0.05	-0.08	0.11	0.30	7.7E-01
C-reactive protein	-0.13	0.07	-0.26	0.00	-1.95	5.2E-02

Supplementary Table S11: Genetic correlation between FMD without top loci with vascular related diseases and traits

rg: genetic correlation, se: standard error, P: P-value for the genetic correlation. eGFR: estimated glomerular filtration rate.

Disease or trait	rg	se	L95_rg	U95_rg	z	P
Hypertension	0.36	0.07	0.22	0.51	4.91	9.3E-07
Systolic Blood Pressure	0.44	0.08	0.29	0.59	5.67	1.4E-08
Diastolic Blood Pressure	0.38	0.07	0.24	0.52	5.39	7.1E-08
Pulse Pressure	0.36	0.07	0.23	0.49	5.40	6.8E-08
Coronary artery disease	-0.10	0.06	-0.22	0.02	-1.57	1.2E-01
Myocardial infarction	-0.10	0.07	-0.23	0.04	-1.40	1.6E-01
Migraine	0.27	0.09	0.10	0.44	3.09	2.0E-03
Any stroke	0.18	0.09	0.01	0.35	2.12	3.4E-02
Any ischemic stroke	0.19	0.08	0.03	0.35	2.33	2.0E-02
Large artery stroke	0.35	0.32	-0.27	0.97	1.09	2.7E-01
Cardioembolic stroke	0.40	0.13	0.15	0.66	3.13	1.7E-03
Small vessel stroke	0.25	0.17	-0.09	0.58	1.43	1.5E-01
Intracranial aneurysm	0.37	0.09	0.19	0.55	4.02	5.8E-05
Subarachnoid haemorrhage	0.37	0.10	0.17	0.57	3.58	3.4E-04
Unruptured intracranial aneurysm	0.33	0.14	0.06	0.60	2.44	1.5E-02
Cervical artery dissection	0.60	0.33	-0.05	1.25	1.82	6.9E-02
High density lipopoteins	-0.03	0.05	-0.12	0.06	-0.57	5.7E-01
Low density lipoproteins	-0.18	0.06	-0.31	-0.06	-2.84	4.5E-03
Total cholesterol	-0.15	0.06	-0.27	-0.03	-2.48	1.3E-02
Tryglicerydes	0.09	0.05	0.00	0.19	1.92	5.4E-02
Apolipoprotein A	0.01	0.05	-0.09	0.10	0.19	8.5E-01
Apolipoprotein B	-0.17	0.06	-0.30	-0.04	-2.64	8.3E-03
Creatinine	0.04	0.05	-0.06	0.14	0.80	4.3E-01
CystatinC	0.02	0.04	-0.06	0.10	0.46	6.5E-01
eGFR creatinine	0.02	0.05	-0.08	0.11	0.37	7.1E-01
eGFR cystatinC	0.07	0.10	-0.12	0.27	0.73	4.6E-01
Urine albumin to creatinin ratio	0.09	0.06	-0.02	0.20	1.56	1.2E-01
Blood urea nitrogen	0.00	0.06	-0.12	0.12	0.01	9.9E-01
Urate	0.01	0.05	-0.08	0.10	0.22	8.2E-01
Urea	0.04	0.05	-0.06	0.14	0.76	4.4E-01
C-reactive protein	-0.09	0.06	-0.21	0.03	-1.47	1.4E-01

Supplementary Table S12: Genetic correlation between FMD and cardiovascular and neurovascular diseases and traits conditionned on systolic blood pressure genetic association data after excluding SNPs in the current 4 genome-wide associated loci.

rg: genetic correlation, se: standard error, P: P-value of genetic correlation/ eGFR: estimated glomerular filtration rate.

**Disease or trait**	**rg**	**se**	**L95_rg**	**U95_rg**	**z**	**P**
Coronary artery disease	-0.30	0.08	-0.46	-0.14	-3.73	2.0E-04
Myocardial infarction	-0.29	0.09	-0.46	-0.12	-3.39	7.0E-04
Migraine	0.32	0.10	0.13	0.51	3.27	1.1E-03
Any stroke	-0.01	0.09	-0.19	0.17	-0.10	9.2E-01
Any ischemic stroke	0.02	0.09	-0.16	0.19	0.18	8.6E-01
Large artery stroke	0.01	0.27	-0.51	0.54	0.06	9.6E-01
Cardioembolic stroke	0.37	0.14	0.10	0.64	2.68	7.4E-03
Small vessel stroke	0.08	0.18	-0.27	0.43	0.45	6.5E-01
Intracranial aneurysm	0.28	0.10	0.09	0.47	2.89	3.8E-03
Subarachnoid haemorrhage	0.27	0.11	0.06	0.49	2.49	1.3E-02
Unruptured intracranial aneurysm	0.26	0.15	-0.03	0.54	1.76	7.9E-02
Cervical artery dissection	0.53	0.37	-0.20	1.25	1.43	1.5E-01
High density lipopoteins	-0.02	0.05	-0.12	0.08	-0.41	6.8E-01
Low density lipoproteins	-0.13	0.07	-0.26	0.01	-1.87	6.2E-02
Total cholesterol	-0.11	0.07	-0.24	0.02	-1.67	9.6E-02
Tryglicerydes	0.06	0.06	-0.05	0.17	1.07	2.9E-01
Apolipoprotein A	-0.01	0.05	-0.12	0.09	-0.28	7.8E-01
Apolipoprotein B	-0.14	0.07	-0.28	-0.01	-2.04	4.1E-02
Creatinine	0.05	0.06	-0.06	0.16	0.91	3.6E-01
CystatinC	0.00	0.05	-0.09	0.09	0.07	9.4E-01
eGFR creatinine	0.03	0.05	-0.08	0.13	0.52	6.0E-01
eGFR cystatinC	0.12	0.11	-0.09	0.34	1.12	2.6E-01
Urine albumin to creatinin ratio	-0.10	0.06	-0.21	0.01	-1.72	8.6E-02
Blood urea nitrogen	-0.03	0.06	-0.15	0.09	-0.49	6.3E-01
Urate	-0.06	0.05	-0.17	0.04	-1.18	2.4E-01
Urea	0.02	0.05	-0.09	0.12	0.29	7.7E-01
C-reactive protein	-0.13	0.07	-0.28	0.01	-1.85	6.4E-02

Supplementary Table S13: **Details of genotyping, pre-imputation and post-imputation quality control steps per study.**
N : number of samples, IBD: Identity by descent , PCA: Principal component analysis. *Nine non-European cases were kept and matched for ctrls by PCs coordinated.

				QC steps						Post imputation		Meta-analysis without correction for λGC per study			Meta-analysis with correction for λGC per study	
**Country**	**cohort**	**status**	**genotyping array**	**N (genotyped)**	**Heterozygosity / Call Rate**	**Relatdness/ Duplication (IBD>0.185)**	**Non European excluded (PCA analysis)**	**Non multifocal or unknown**	**N included in GWAS**	**N SNPs**	**λGC**	**N SNPs**	**λGC**	**LDSR intercept**	**λGC**	**LDSR intercept**
France	ARCADIA	Cases	Infinium OmniExpressExome-8v1.3	786	27	59	54	215	431	6810391	1.02	5483710	1.04	1.03	1.05	1.04
	3 Cities study	controls	Illumina-Human660W-Quad v1.0	1487	0	0	0	NR	1487							
POL	ARCADIA-POL	Cases	Illumina-Infinium Global Screening Array-24 v2.0 +MD	129	0	4	0	18	107	6450082	0.96					
	WOBASZ II	controls		298	3	0	0	NR	295							
Europe	FEIRI	Cases	Illumina-Infinium Global Screening Array-24 v2.0 +MD	374	7	4	19	101	243	6815826	1.03					
	ASKLEPIOS	controls		658	9	32	2	NR	615							
USA	MayoVDB	Cases	Illumina-Infinium-Human-Core-Exome	175	3	3	0	53	116	6697599	0.97					
	MayoVDB	controls		1177	16	20	0	NR	1141							
USA	DEFINE-FMD	Cases	Illumina-Human-Omni-Express-Exome	134	1	7	18	0	108	6567998	0.95					
	DEFINE-FMD	controls		164	5	8	25	NR	126							
USA	UM/CCF-FMD	Cases	illumina-Infinium CoreExome-24v1.1 BeadArray with 607,778 SNP markers (UM_HUNT_Biobank_v1-1_20006200_A)	584	0	11	0*	22	551	6818332	1.01					
	UM/CCF-MGI	controls		3438	0	2	0*	NR	3436							

Supplementary Table S14: Epigenomic datasets used for annotations of FMD associated variants.

ID	Type	Target	tissue	Origin
HCtASMC-A	ATAC-Seq		carotid artery	This study
HCtASMC-B	ATAC-Seq		carotid artery	This study
HCASMC-A	ATAC-Seq		coronary artery	This study
HCtAEC-A	ATAC-Seq		carotid artery	This study
HCtAEC-B	ATAC-Seq		carotid artery	This study
HCAEC	ATAC-Seq		coronary artery	This study
HDF-A	ATAC-Seq		dermis	This study
HDF-B	ATAC-Seq		dermis	This study
HCF	ATAC-Seq		heart	This study
NCA-A	ATAC-Seq		coronary artery	https://www.ncbi.nlm.nih.gov/sra/?term=SRR2378591
NCA-B	ATAC-Seq		coronary artery	https://www.ncbi.nlm.nih.gov/sra/?term=SRR2378592
NCA-C	ATAC-Seq		coronary artery	https://www.ncbi.nlm.nih.gov/sra/?term=SRR2378593
ENCFF874DSY	ChIP-Seq	H3K27ac	coronary artery	https://www.encodeproject.org/files/ENCFF874DSY/@@download/ENCFF874DSY.bed.gz
ENCFF595RQJ	ChIP-Seq	H3K27ac	thoracic aorta	https://www.encodeproject.org/files/ENCFF595RQJ/@@download/ENCFF595RQJ.bed.gz
ENCFF208DZK	ChIP-Seq	H3K27ac	ascending aorta	https://www.encodeproject.org/files/ENCFF208DZK/@@download/ENCFF208DZK.bed.gz
ENCFF722XLA	ChIP-Seq	H3K27ac	coronary artery	https://www.encodeproject.org/files/ENCFF722XLA/@@download/ENCFF722XLA.bed.gz
ENCFF776MXU	ChIP-Seq	H3K27ac	tibial artery	https://www.encodeproject.org/files/ENCFF776MXU/@@download/ENCFF776MXU.bed.gz
ENCFF121HDP	ChIP-Seq	H3K27ac	tibial artery	https://www.encodeproject.org/files/ENCFF121HDP/@@download/ENCFF121HDP.bed.gz
ENCFF028BAF	ChIP-Seq	H3K27ac	tibial artery	https://www.encodeproject.org/files/ENCFF028BAF/@@download/ENCFF028BAF.bed.gz
ENCFF030AUO	ChIP-Seq	H3K27ac	thoracic aorta	https://www.encodeproject.org/files/ENCFF030AUO/@@download/ENCFF030AUO.bed.gz
ENCFF003NBP	ChIP-Seq	H3K27ac	thoracic aorta	https://www.encodeproject.org/files/ENCFF003NBP/@@download/ENCFF003NBP.bed.gz
ENCFF967DLF	ChIP-Seq	H3K27ac	thoracic aorta	https://www.encodeproject.org/files/ENCFF967DLF/@@download/ENCFF967DLF.bed.gz
ENCFF134QRV	ChIP-Seq	H3K27ac	tibial artery	https://www.encodeproject.org/files/ENCFF134QRV/@@download/ENCFF134QRV.bed.gz
ENCFF020COG	ChIP-Seq	H3K27ac	ascending aorta	https://www.encodeproject.org/files/ENCFF020COG/@@download/ENCFF020COG.bed.gz
ENCFF316LHC	ChIP-Seq	H3K4me1	tibial artery	https://www.encodeproject.org/files/ENCFF316LHC/@@download/ENCFF316LHC.bed.gz
ENCFF914DAA	ChIP-Seq	H3K4me1	tibial artery	https://www.encodeproject.org/files/ENCFF914DAA/@@download/ENCFF914DAA.bed.gz
ENCFF974YFN	ChIP-Seq	H3K4me1	tibial artery	https://www.encodeproject.org/files/ENCFF974YFN/@@download/ENCFF974YFN.bed.gz
ENCFF980YJQ	ChIP-Seq	H3K4me1	tibial artery	https://www.encodeproject.org/files/ENCFF980YJQ/@@download/ENCFF980YJQ.bed.gz
ENCFF256LHH	ChIP-Seq	H3K4me1	tibial artery	https://www.encodeproject.org/files/ENCFF256LHH/@@download/ENCFF256LHH.bed.gz
ENCFF194TWC	ChIP-Seq	H3K4me1	ascending aorta	https://www.encodeproject.org/files/ENCFF194TWC/@@download/ENCFF194TWC.bed.gz
ENCFF340GNE	ChIP-Seq	H3K4me1	thoracic aorta	https://www.encodeproject.org/files/ENCFF340GNE/@@download/ENCFF340GNE.bed.gz
ENCFF080KQQ	ChIP-Seq	H3K4me1	thoracic aorta	https://www.encodeproject.org/files/ENCFF080KQQ/@@download/ENCFF080KQQ.bed.gz
ENCFF831ZKB	ChIP-Seq	H3K4me1	thoracic aorta	https://www.encodeproject.org/files/ENCFF831ZKB/@@download/ENCFF831ZKB.bed.gz
ENCFF821NNE	ChIP-Seq	H3K4me1	tibial artery	https://www.encodeproject.org/files/ENCFF821NNE/@@download/ENCFF821NNE.bed.gz
ENCFF651FRA	ChIP-Seq	H3K4me1	thoracic aorta	https://www.encodeproject.org/files/ENCFF651FRA/@@download/ENCFF651FRA.bed.gz
ENCFF633GZP	ChIP-Seq	H3K4me1	ascending aorta	https://www.encodeproject.org/files/ENCFF633GZP/@@download/ENCFF633GZP.bed.gz
ENCFF154GIE	ChIP-Seq	H3K4me3	coronary artery	https://www.encodeproject.org/files/ENCFF154GIE/@@download/ENCFF154GIE.bed.gz
ENCFF682FCZ	ChIP-Seq	H3K4me3	coronary artery	https://www.encodeproject.org/files/ENCFF682FCZ/@@download/ENCFF682FCZ.bed.gz
ENCFF608ZGR	ChIP-Seq	H3K4me3	coronary artery	https://www.encodeproject.org/files/ENCFF608ZGR/@@download/ENCFF608ZGR.bed.gz
ENCFF682ELX	ChIP-Seq	H3K4me3	ascending aorta	https://www.encodeproject.org/files/ENCFF682ELX/@@download/ENCFF682ELX.bed.gz
ENCFF647QUI	ChIP-Seq	H3K4me3	tibial artery	https://www.encodeproject.org/files/ENCFF647QUI/@@download/ENCFF647QUI.bed.gz
ENCFF313MVM	ChIP-Seq	H3K4me3	tibial artery	https://www.encodeproject.org/files/ENCFF313MVM/@@download/ENCFF313MVM.bed.gz
ENCFF701WDJ	ChIP-Seq	H3K4me3	tibial artery	https://www.encodeproject.org/files/ENCFF701WDJ/@@download/ENCFF701WDJ.bed.gz
ENCFF817XTD	ChIP-Seq	H3K4me3	tibial artery	https://www.encodeproject.org/files/ENCFF817XTD/@@download/ENCFF817XTD.bed.gz
ENCFF205QFR	ChIP-Seq	H3K4me3	thoracic aorta	https://www.encodeproject.org/files/ENCFF205QFR/@@download/ENCFF205QFR.bed.gz
ENCFF582FDJ	ChIP-Seq	H3K4me3	thoracic aorta	https://www.encodeproject.org/files/ENCFF582FDJ/@@download/ENCFF582FDJ.bed.gz
ENCFF946FND	ChIP-Seq	H3K4me3	thoracic aorta	https://www.encodeproject.org/files/ENCFF946FND/@@download/ENCFF946FND.bed.gz
ENCFF930ZZM	ChIP-Seq	H3K4me3	ascending aorta	https://www.encodeproject.org/files/ENCFF930ZZM/@@download/ENCFF930ZZM.bed.gz
ENCFF593YQY	ChIP-Seq	H3K4me3	thoracic aorta	https://www.encodeproject.org/files/ENCFF593YQY/@@download/ENCFF593YQY.bed.gz

Supplementary Table S15: details of summary statistics used in to calculate the genetic correlation with FMD
for continuous traits Neff= sample size of study
for binary traits Neff was calculated as follow : neff= 4/(1/Ncases+1/Nctrls)
* : range.

Study	groupe of traits	trait	Type	Neff	N Cases	N controls	source
UK BioBank	Blood pressure	Hypertension (HTN)	binary	277286	93560	267581	http://www.nealelab.is/uk-biobank
Evangelou et al	Blood pressure	Systolic Blood Pressure (SBP)	continuous	[637578 - 745820]*	-	-	ftp://ftp.ebi.ac.uk/pub/databases/gwas/summary_statistics/EvangelouE_30224653_GCST006624
Evangelou et al	Blood pressure	Diastolic Blood Pressure (DBP)	continuous	[647307 - 757601]*	-	-	ftp://ftp.ebi.ac.uk/pub/databases/gwas/summary_statistics/EvangelouE_30224653_GCST006630
Evangelou et al	Blood pressure	Pulse Pressure (PP)	continuous	[586460 - 745787]*	-	-	ftp://ftp.ebi.ac.uk/pub/databases/gwas/summary_statistics/EvangelouE_30224653_GCST006629
CARDIoGRAMplusC4D Consortium	Artery disease	Coronary Artery Disease (CAD)	binary	162973	60801	123504	http://www.cardiogramplusc4d.org/data-downloads/
CARDIoGRAMplusC4D Consortium	Artery disease	Myocardial Infarction (MI)	binary	126612	42561	123504	http://www.cardiogramplusc4d.org/data-downloads/
UK BioBank	Neurovascular	Migraine	binary	41332	10647	350494	http://www.nealelab.is/uk-biobank
MEGASTROKE consortium	Stroke	Any stroke (AS)	binary	147590	40585	406111	https://www.megastroke.org/
MEGASTROKE consortium	Stroke	Any Ischemic Stroke (AIS)	binary	126232	34217	406111	https://www.megastroke.org/
MEGASTROKE consortium	Stroke	Large Artery Stroke (LAS)	binary	17306	4373	406111	https://www.megastroke.org/
MEGASTROKE consortium	Stroke	Cardioembolic Stroke (CES)	binary	28271	7193	406111	https://www.megastroke.org/
MEGASTROKE consortium	Stroke	Small Vessel Stroke (SVS)	binary	21262	5386	406111	https://www.megastroke.org/
Mark K Bakker et al	Aneurysm	Intracranial Aneurysm (IA)	binary	[14576 - 24253]*	-	-	DOI: 10.1038/s41588-020-00725-7
Mark K Bakker et al	Aneurysm	Subarachnoid Haemorrhage (SAH)	binary	[10203 - 17019]*	-	-	DOI: 10.1038/s41588-020-00725-7
Mark K Bakker et al	Aneurysm	unruptured Intracranial Aneurysm (uIA)	binary	[5225 - 7721]*	-	-	DOI: 10.1038/s41588-020-00725-7
Stéphanie Debette et al	Dissection	Cervical Artery Dissection (CeAD)	binary	5081	1393	14416	DOI: 10.1038/ng.3154
UK BioBank	Blood lipids	HDL	continuous	315133	-	-	http://www.nealelab.is/uk-biobank
UK BioBank	Blood lipids	LDL	continuous	343621	-	-	http://www.nealelab.is/uk-biobank
UK BioBank	Blood lipids	Total cholesterol (TC)	continuous	344278	-	-	http://www.nealelab.is/uk-biobank
UK BioBank	Blood lipids	Trygliceryde (TG)	continuous	343992	-	-	http://www.nealelab.is/uk-biobank
UK BioBank	Blood lipids	Apolipoprotein A (ApoA)	continuous	313387	-	-	http://www.nealelab.is/uk-biobank
UK BioBank	Blood lipids	Apolipoprotein B (ApoB)	continuous	342590	-	-	http://www.nealelab.is/uk-biobank
UK BioBank	Renal function	Creatinine	continuous	344104	-	-	http://www.nealelab.is/uk-biobank
UK BioBank	Renal function	CystatinC	continuous	344264	-	-	http://www.nealelab.is/uk-biobank
CKDgen Consortium	Renal function	eGFR creatinine	continuous	[378166 - 567460]*	-	-	https://ckdgen.imbi.uni-freiburg.de
CKDgen Consortium	Renal function	eGFR cystatinC	continuous	[16097 - 24061]*	-	-	https://ckdgen.imbi.uni-freiburg.de
CKDgen Consortium	Renal function	UACR	continuous	[471173 - 547361]*	-	-	https://ckdgen.imbi.uni-freiburg.de
CKDgen Consortium	Renal function	BUN	continuous	[161936 - 243031]*	-	-	https://ckdgen.imbi.uni-freiburg.de
UK BioBank	Renal function	Urate	continuous	343836	-	-	http://www.nealelab.is/uk-biobank
UK BioBank	Renal function	Urea	continuous	344052	-	-	http://www.nealelab.is/uk-biobank
UK BioBank	Renal function	CRP	continuous	343524	-	-	http://www.nealelab.is/uk-biobank

Assessment of Renal and Cervical Artery Dysplasia (ARCADIA) investigators

Investigators by recruitment center, city and (number of patients enrolled)

- Hôpital Européen Georges Pompidou, Paris (156): Arshid Azarine, Michel Azizi, Gilles Chatellier, Antoine Chedid, Béatrice Fiquet, Xavier Jeunemaitre, Elie Mousseaux, Pierre-François Plouin;
- Centre hospitalo-universitaire Grenoble (140): Jean-Philippe Baguet, Olivier Ormezzano, Frédéric Thony;
- Centre hospitalo-universitaire Timone, Marseille (37): François Silhol;
- Centre hospitalier Sainte-Anne, Paris (28): Eric Bodiguel, Valérie Domigo, Catherine Oppenheim, Marta Pasquini, Emmanuel Touzé, Denis Trystram;
- Centre hospitalo-universitaire Clermont-Ferrand (28): Louis Boyer, Pierre Clavelou;
- Centre hospitalo-universitaire Rangueil, Toulouse (25): Bernard Chamontin, Béatrice Duly-Bouhanick;
- Hôpital Cardiologique, Lille (21): Hilde Hénon, Claire Mounier-Vehier;
- Cliniques Universitaires Saint-Luc, Brussels (19): Parla Astarci, Pierre Goffette, Frank Hammer, Jean-Philippe Lengelé, Alexandre Persu, Francesca Severino, Robert Verhelst;
- Centre hospitalo-universitaire de Caen (10): Claire Le Hello;
- Hôpital Saint André, Bordeaux (10): Philippe Gosse;
- Centre hospitalo-universitaire Lariboisière, Paris (6): Dominique Hervé;
- Centre hospitalier de Versailles (3): Fernando Pico;
- Centre hospitalo-universitaire de Nancy (2): Michèle Kessler, Patrick Rossignol;
- Centre hospitalo-universitaire de la Pitié Salpêtrière, Paris (2): Yves Sanson;
- Hôpital Saint Joseph, Paris (2): Mathieu Zuber;
- Hôpital Bichat, Paris (2): Mikael Mazighi;
- Hôpital Tenon, Paris (1): Sonia Alamowitch.
- Imaging Committee of the study, Hôpital Européen Georges Pompidou, Paris: Arshid Azarine, Elie Mousseaux (chair), Catherine Oppenheim, Pierre-François Plouin, Emmanuel Touzé, Frédéric Thony.

Investigators of the European/International FMD Registry and Initiative (FEIRI) who contributed to this study.

Belgium: Alexandre Persu, Marco Pappaccogli, Christophe Beauloye, Patrick Chenu, Frank Hammer, Pierre Goffette, Parla Astarci, André Peeters, Robert Verhelst and Miikka Vikkula (Cliniques Universitaires Saint-Luc, Brussels); Patricia Van der Niepen and Frank Van Tussenbroek (Universitair Ziekenhuis Brussel, Brussels); Tine De Backer, Sofie Gevaert, Dimitri Hemelsoet and Luc Defreyne (Universitair Ziekenhuis Gent, Gent); Hilde Heuten, Laetitia Yperzeele and Thijs Van der Zijden (Universitair Ziekenhuis Antwerpen, Antwerpen); Jean-Philippe Lengelé (Grand Hôpital De Charleroi, Charleroi); Jean-Marie Krzesinski and Muriel Sprynger (CHU Sart-Tilman, Liège); Philippe Delmotte (Hopitâl Ambroise Paré, Mons); Peter Verhamme and Thomas Vanassche (UZ-Gasthuisberg, Leuven); Pasquale Scoppettuolo and Jean-Claude Wautrecht (University Hospital ULB Erasme, Brussels, Belgium); Wouter Vinck (GZA ziekenhuizen – campus Sint-Augustinus, Wilrijk).

Croatia: Bojan Jelaković and Zivka Dika (Department of Nephrology, Arterial Hypertension, Dialysis and Transplantation, University Hospital Center Zagreb, School of Medicine University of Zagreb, Zagreb, Croatia)

Finland: Daniel Gordin, Ilkka Tikkanen, Maarit Venermo and RN Anita Mäkelä (Helsinki University Hospital, Helsinki, Finland)

France: Laurent Toubiana (FEIRI platform- Sorbonne Université, Université Paris 6, Sorbonne Paris Cité, INSERM, UMR_S1142, LIMICS ; IRSAN Research Institute for the valorization of health data, Paris, France).

Germany: Felix Mahfoud, Juliane Dederer and Saarraaken Kulenthiran (Saarland University Hospital, Homburg/Saar)

Ireland: Caitriona Canning (St. James's Hospital, Dublin, Ireland)

Italy: Rosa Maria Bruno, Stefano Taddei and Alessandra Bacca (Department of Clinical and Experimental Medicine University of Pisa, Pisa), Ilaria Petrucci (Sant'Anna School of Advanced Studies, Pisa); Franco Rabbia, Marco Pappaccogli and Silvia Di Monaco (AOU Città della Salute e della Scienza, Torino); Gian Paolo Rossi, Silvia Lerco, Angiola Bolis, Laura Zotta and Livia Lenzini (University Hospital, Padova); Pietro Minuz, Giancarlo Mansueto, Sergio De Marchi, Denise Marcon (Università di Verona, Verona).

The Netherlands: Daan J van Twist (Department of Internal Medicine, Zuyderland Medical Centre, Sittard/Heerlen), Bram Kroon and Peter de Leeuw (Maastricht University Medical Center, Maastricht); Wilko Spiering (University Medical Center Utrecht, Utrecht); Bert-Jan van den Born (Academic Medical Centre, University of Amsterdam, Amsterdam)

Spain: Juan Diego Mediavilla and Fernando Jaen Aguila (Virgen of The Nieves University Hospital, Granada, Spain); Nicolas Roberto Robles (Hospital Infanta Cristina, Badajoz); Esteban Poch, Enrique Montagud-Marrahi, Alicia Molina, Elena Guillen (Department of Nephrology and Kidney Transplantation, University of Barcelona, Barcelona) and Marta Burrel (Department of Radiology, Hospital Clinic, Barcelona)
Switzerland: Gregor Wuerzner (Service of Nephrology and Hypertension, Department of Medicine Lausanne University Hospital – CHUV, Lausanne); Lucia Mazzolai and Giacomo Buso (Division of Angiology, Heart and Vessel Department, Lausanne University Hospital - CHUV, Centre of rare vascular diseases and RAVAD registry, Lausanne).
Tunisia: Faiçal Jarraya and Hanen Chaker (Service de Néphrologie, CHU Hédi Chaker, Sfax, Tunisie)

International stroke genetics consortium (ISGC) intracranial aneurysm working group

Masato Akiyama
Laboratory for Statistical and Translational Genetics, RIKEN Center for Integrative Medical Sciences, Yokohama, Japan.
Department of Ophthalmology, Graduate School of Medical Sciences, Kyushu University, Fukuoka, Japan.
Department of Ocular Pathology and Imaging Science, Graduate School of Medical Sciences, Kyushu University, Fukuoka, Japan.

Varinder S. Alg
Stroke Research Centre, University College London, Institute of Neurology, London, UK

Marianne Bakke Johnsen
K. G. Jebsen Center for Genetic Epidemiology, Department of Public Health and Nursing, Faculty of Medicine and Health Sciences, Norwegian University of Science and Technology, Trondheim, Norway.
Research and Communication Unit for Musculoskeletal Health (FORMI), Department of Research, Innovation and Education, Division of Clinical Neuroscience, Oslo University Hospital, Oslo, Norway.
Institute of Clinical Medicine, Faculty of Medicine, University of Oslo, Oslo, Norway.

Mark K. Bakker
Department of Neurology and Neurosurgery, University Medical Center Utrecht Brain Center, Utrecht University, Utrecht, The Netherlands

Philippe Bijlenga
Neurosurgery Division, Department of Clinical Neurosciences, Faculty of Medicine, Geneva University Hospitals, Geneva, Switzerland.

Sigrid Børte
K. G. Jebsen Center for Genetic Epidemiology, Department of Public Health and Nursing, Faculty of Medicine and Health Sciences, Norwegian University of Science and Technology, Trondheim, Norway.
Research and Communication Unit for Musculoskeletal Health (FORMI), Department of Research, Innovation and Education, Division of Clinical Neuroscience, Oslo University Hospital, Oslo, Norway.
Institute of Clinical Medicine, Faculty of Medicine, University of Oslo, Oslo, Norway.

Romain Bourcier
Université de Nantes, CHU Nantes, INSERM, CNRS, l'institut du thorax, Nantes, France.
CHU Nantes, Department of Neuroradiology, Nantes, France.

Joseph P. Broderick
University of Cincinnati College of Medicine, Cincinnati, OH, USA.

Ben M. Brumpton
K. G. Jebsen Center for Genetic Epidemiology, Department of Public Health and Nursing, Faculty of Medicine and Health Sciences, Norwegian University of Science and Technology, Trondheim, Norway.

Zhengming Chen
Clinical Trial Service Unit and Epidemiological Studies Unit, Nuffield Department of Population Health, University of Oxford, Oxford, U.K.

Jerome Dauvillier
SIB Swiss Institute of Bioinformatics.

Hubert Desal
Université de Nantes, CHU Nantes, INSERM, CNRS, l'institut du thorax, Nantes, France.
CHU Nantes, Department of Neuroradiology, Nantes, France.

Christian Dina
Université de Nantes, CHU Nantes, INSERM, CNRS, l'institut du thorax, Nantes, France.

François Eugène
Department of Neuroradiology, University hospital of Rennes, Rennes, France.

Mikael von Und Zu Fraunberg
Neurosurgery NeuroCenter Kuopio University Hospital Kuopio Finland.
Institute of Clinical Medicine Faculty of Health Sciences University of Eastern Finland Kuopio Finland.

Christoph M. Friedrich
University of Applied Science and Arts, Dortmund.

Emília I. Gaál-Paavola
Department of Neurosurgery, Helsinki University Hospital, University of Helsinki, Finland.
Clinical Neurosciences, University of Helsinki, Topeliuksenkatu 5, 00260, Helsinki, Finland.

Jean-Christophe Gentric
Department of Neuroradiology, University hospital of Brest, Brest, France.

Sven Hirsch
Zurich University of Applied Sciences, School of Life Sciences and Facility Management.

Isabel C. Hostettler
Stroke Research Centre, University College London Queen Square Institute of Neurology, London, UK
Department of Neurosurgery, Klinikum rechts der Isar, Technical University Munich, Munich, Germany.

Henry Houlden
Neurogenetics Laboratory, The National Hospital of Neurology and Neurosurgery, London, UK.

Kristian Hveem
K. G. Jebsen Center for Genetic Epidemiology, Department of Public Health and Nursing, Faculty of Medicine and Health Sciences, Norwegian University of Science and Technology, Trondheim, Norway.
HUNT Research Center, Department of Public Health and Nursing, Faculty of Medicine and Health Sciences, Norwegian University of Science and Technology, Trondheim, Norway.

Juha E. Jääskeläinen
Neurosurgery NeuroCenter Kuopio University Hospital Kuopio Finland.
Institute of Clinical Medicine Faculty of Health Sciences University of Eastern Finland Kuopio Finland.

Yoichiro Kamatani
Graduate School of Frontier Sciences, The University of Tokyo, Tokyo, Japan.

Masaru Koido
Laboratory for Statistical and Translational Genetics, RIKEN Center for Integrative Medical Sciences, Yokohama, Japan.
Department of Cancer Biology, Institute of Medical Science, The University of Tokyo, Tokyo, Japan.

Liming Li
School of Public Health, Peking University Health Science Center, Beijing, China.

Kuang Lin
Clinical Trial Service Unit and Epidemiological Studies Unit, Nuffield Department of Population Health, University of Oxford, Oxford, U.K.

Antti Lindgren
Neurosurgery NeuroCenter Kuopio University Hospital Kuopio Finland.
Institute of Clinical Medicine Faculty of Health Sciences University of Eastern Finland Kuopio Finland.

Olivier Martin
SIB Swiss Institute of Bioinformatics.

Koichi Matsuda
Graduate School of Frontier Sciences, The University of Tokyo, Tokyo, Japan.

Iona Y. Millwood
Clinical Trial Service Unit and Epidemiological Studies Unit, Nuffield Department of Population Health, University of Oxford, Oxford, U.K.
Medical Research Council Population Health Research Unit, University of Oxford, Oxford, U.K.

Sandrine Morel
Department of Pathology and Immunology, Faculty of Medicine, University of Geneva, Geneva, Switzerland; Neurosurgery Division, Department of Clinical Neurosciences, Faculty of Medicine, Geneva University Hospitals, Geneva, Switzerland.

Olivier Naggara
Pediatric Radiology, Necker Hospital for Sick Children, Université Paris Descartes, Paris, France.
Department of Neuroradiology, Sainte-Anne Hospital and Université Paris Descartes, INSERM UMR S894, Paris, France.

Mika Niemelä
Department of Neurosurgery, Helsinki University Hospital, University of Helsinki, Finland.

Joanna Pera
Department of Neurology, Faculty of Medicine, Jagiellonian University Medical College, ul. Botaniczna 3, 31-503, Krakow, Poland.

Kristiina Rannikmäe
Centre for Medical Informatics, Usher Institute, University of Edinburgh, Edinburgh, UK.

Guy A. Rouleau
Montreal Neurological Institute and Hospital, McGill University, Montréal, QC, Canada.

Marie Søfteland Sandvei
Department of Public Health and Nursing, Faculty of Medicine and Health Sciences, Norwegian University of Science and Technology, Trondheim, Norway.
The Cancer Clinic, St Olavs Hospital, Trondheim University Hospital, Trondheim, Norway.

Sabine Schilling
Zurich University of Applied Sciences, School of Life Sciences and Facility Management.

Eimad Shotar
Department of Neuroradiology, Pitié-Salpêtrière Hospital, Paris, France.

Agnieszka Slowik

Department of Neurology, Faculty of Medicine, Jagiellonian University Medical College, ul. Botaniczna 3, 31-503, Krakow, Poland.

Cathie L.M. Sudlow
Centre for Medical Informatics, Usher Institute, University of Edinburgh, Edinburgh, UK.
UK Biobank, Cheadle, Stockport, UK

Richard Redon
Université de Nantes, CHU Nantes, INSERM, CNRS, l'institut du thorax, Nantes, France.

Gabriel J.E. Rinkel
Department of Neurology and Neurosurgery, University Medical Center Utrecht Brain Center, Utrecht University, Utrecht, The Netherlands

Ynte M. Ruigrok
Department of Neurology and Neurosurgery, University Medical Center Utrecht Brain Center, Utrecht University, Utrecht, The Netherlands

Robin G. Walters
Clinical Trial Service Unit and Epidemiological Studies Unit, Nuffield Department of Population Health, University of Oxford, Oxford, U.K.

David J. Werring
Stroke Research Centre, University College London Queen Square Institute of Neurology, London, UK.

Cristen J. Willer
Department of Internal Medicine, Division of Cardiovascular Medicine, University of Michigan, Ann Arbor, 48109, MI, USA.

Bendik S. Winsvold
Department of Research, Innovation and Education, Division of Clinical Neuroscience, Oslo University Hospital, Oslo, Norway.
K. G. Jebsen Center for Genetic Epidemiology, Department of Public Health and Nursing, Faculty of Medicine and Health Sciences, Norwegian University of Science and Technology, Trondheim, Norway.

Daniel Woo
University of Cincinnati College of Medicine, Cincinnati, OH, USA.

Bradford B. Worrall
Departments of Neurology and Public Health Sciences, University of Virginia School of Medicine, Charlottesville, VA, USA.

Sirui Zhou
Lady Davis Institute, Jewish General Hospital, McGill University, Montréal, QC, Canada.

John-Anker Zwart
Department of Research, Innovation and Education, Division of Clinical Neuroscience, Oslo University Hospital, Oslo, Norway.
K. G. Jebsen Center for Genetic Epidemiology, Department of Public Health and Nursing, Faculty of Medicine and Health Sciences, Norwegian University of Science and Technology, Trondheim, Norway.
Institute of Clinical Medicine, Faculty of Medicine, University of Oslo, Oslo, Norway.

MEGASTROKE CONSORTIUM

Rainer Malik [1], Ganesh Chauhan [2], Matthew Traylor [3], Muralidharan Sargurupremraj [4,5], Yukinori Okada [6,7,8], Aniket Mishra [4,5], Loes Rutten-Jacobs [3], Anne-Katrin Giese [9], Sander W van der Laan [10], Solveig Gretarsdottir [11], Christopher D Anderson [12,13,14,14], Michael Chong [15], Hieab HH Adams [16,17], Tetsuro Ago [18], Peter Almgren [19], Philippe Amouyel [20,21], Hakan Ay [22,13], Traci M Bartz [23], Oscar R Benavente [24], Steve Bevan [25], Giorgio B Boncoraglio [26], Robert D Brown, Jr. [27], Adam S Butterworth [28,29], Caty Carrera [30,31], Cara L Carty [32,33], Daniel I Chasman [34,35], Wei-Min Chen [36], John W Cole [37], Adolfo Correa [38], Ioana Cotlarciuc [39], Carlos Cruchaga [40,41], John Danesh [28,42,43,44], Paul IW de Bakker [45,46], Anita L DeStefano [47,48], Marcel den Hoed [49], Qing Duan [50], Stefan T Engelter [51,52], Guido J Falcone [53,54], Rebecca F Gottesman [55], Raji P Grewal [56], Vilmundur Gudnason [57,58], Stefan Gustafsson [59], Jeffrey Haessler [60], Tamara B Harris [61], Ahamad Hassan [62], Aki S Havulinna [63,64], Susan R Heckbert [65], Elizabeth G Holliday [66,67], George Howard [68], Fang-Chi Hsu [69], Hyacinth I Hyacinth [70], M Arfan Ikram [16], Erik Ingelsson [71,72], Marguerite R Irvin [73], Xueqiu Jian [74], Jordi Jiménez-Conde [75], Julie A Johnson [76,77], J Wouter Jukema [78], Masahiro Kanai [6,7,79], Keith L Keene [80,81], Brett M Kissela [82], Dawn O Kleindorfer [82], Charles Kooperberg [60], Michiaki Kubo [83], Leslie A Lange [84], Carl D Langefeld [85], Claudia Langenberg [86], Lenore J Launer [87], Jin-Moo Lee [88], Robin Lemmens [89,90], Didier Leys [91], Cathryn M Lewis [92,93], Wei-Yu Lin [28,94], Arne G Lindgren [95,96], Erik Lorentzen [97], Patrik K Magnusson [98], Jane Maguire [99], Ani Manichaikul [36], Patrick F McArdle [100], James F Meschia [101], Braxton D Mitchell [100,102], Thomas H Mosley [103,104], Michael A Nalls [105,106], Toshiharu Ninomiya [107], Martin J O'Donnell [15,108], Bruce M Psaty [109,110,111,112], Sara L Pulit [113,45], Kristiina Rannikmäe [114,115], Alexander P Reiner [65,116], Kathryn M Rexrode [117], Kenneth Rice [118], Stephen S Rich [36], Paul M Ridker [34,35], Natalia S Rost [9,13], Peter M Rothwell [119], Jerome I Rotter [120,121], Tatjana Rundek [122], Ralph L Sacco [122], Saori Sakaue [7,123], Michele M Sale [124], Veikko Salomaa [63], Bishwa R Sapkota [125], Reinhold Schmidt [126], Carsten O Schmidt [127], Ulf Schminke [128], Pankaj Sharma [39], Agnieszka Slowik [129], Cathie LM Sudlow [114,115], Christian Tanislav [130], Turgut Tatlisumak [131,132], Kent D Taylor [120,121], Vincent NS Thijs [133,134], Gudmar Thorleifsson [11], Unnur Thorsteinsdottir [11], Steffen Tiedt [1], Stella Trompet [135], Christophe Tzourio [5,136,137], Cornelia M van Duijn [138,139], Matthew Walters [140], Nicholas J Wareham [86], Sylvia Wassertheil-Smoller [141], James G Wilson [142], Kerri L Wiggins [109], Qiong Yang [47], Salim Yusuf [15], Najaf Amin [16], Hugo S Aparicio [185,48], Donna K Arnett [186], John Attia [187], Alexa S Beiser [47,48], Claudine Berr [188], Julie E Buring [34,35], Mariana Bustamante [189], Valeria Caso [190], Yu-Ching Cheng [191], Seung Hoan Choi [192,48], Ayesha Chowhan [185,48], Natalia Cullell [31], Jean-François Dartigues [193,194], Hossein Delavaran [95,96], Pilar Delgado [195], Marcus Dörr [196,197], Gunnar Engström [19], Ian Ford [198], Wander S Gurpreet [199], Anders Hamsten [200,201], Laura Heitsch [202], Atsushi Hozawa [203], Laura Ibanez [204], Andreea Ilinca [95,96], Martin Ingelsson [205], Motoki Iwasaki [206], Rebecca D Jackson [207], Katarina Jood [208], Pekka Jousilahti [63], Sara Kaffashian [4,5], Lalit Kalra [209], Masahiro Kamouchi [210], Takanari Kitazono [211], Olafur Kjartansson [212], Manja Kloss [213], Peter J Koudstaal [214], Jerzy Krupinski [215], Daniel L Labovitz [216], Cathy C Laurie [118], Christopher R Levi [217], Linxin Li [218], Lars Lind [219], Cecilia M Lindgren [220,221], Vasileios Lioutas [222,48], Yong Mei Liu [223], Oscar L Lopez [224], Hirata Makoto [225], Nicolas Martinez-Majander [172], Koichi Matsuda [225], Naoko Minegishi [203], Joan Montaner [226], Andrew P Morris [227,228], Elena Muiño [31], Martina Müller-Nurasyid [229,230,231], Bo Norrving [95,96], Soichi Ogishima [203], Eugenio A Parati [232], Leema Reddy Peddareddygari [56], Nancy L Pedersen [98,233], Joanna Pera [129], Markus Perola [63,234], Alessandro Pezzini [235], Silvana Pileggi [236], Raquel Rabionet [237], Iolanda Riba-Llena [30], Marta Ribasés [238], Jose R Romero [185,48], Jaume Roquer [239,240], Anthony G Rudd [241,242], Antti-Pekka Sarin [243,244], Ralhan Sarju [199], Chloe Sarnowski [47,48], Makoto Sasaki [245], Claudia L Satizabal [185,48], Mamoru Satoh [245], Naveed Sattar [246], Norie Sawada [206], Gerli Sibolt [172], Ásgeir Sigurdsson [247], Albert Smith [248], Kenji Sobue [245], Carolina Soriano-Tárraga [240], Tara Stanne [249], O Colin Stine [250], David J Stott [251], Konstantin Strauch [229,252], Takako Takai [203], Hideo Tanaka [253,254], Kozo Tanno [245], Alexander Teumer [255], Liisa Tomppo [172], Nuria P Torres-Aguila [31], Emmanuel Touze [256,257], Shoichiro Tsugane [206], Andre G Uitterlinden [258], Einar M Valdimarsson [259], Sven J van der Lee [16], Henry Völzke [255], Kenji Wakai [253], David Weir [260], Stephen R Williams [261], Charles DA Wolfe [241,242], Quenna Wong [118], Huichun Xu [191], Taiki Yamaji [206], Dharambir K Sanghera [125,169,170], Olle Melander [19], Christina Jern [171], Daniel Strbian [172,173], Israel Fernandez-Cadenas [31,30], W T Longstreth, Jr [174,65], Arndt Rolfs [175], Jun Hata [107], Daniel Woo [82], Jonathan Rosand [12,13,14], Guillaume Pare [15], Jemma C Hopewell [176], Danish Saleheen [177], Kari Stefansson [11,178], Bradford B

Worrall [179], Steven J Kittner [37], Sudha Seshadri [180,48], Myriam Fornage [74,181], Hugh S Markus [3], Joanna MM Howson [28], Yoichiro Kamatani [6,182], Stephanie Debette [4,5], Martin Dichgans [1,183,184]

1 Institute for Stroke and Dementia Research (ISD), University Hospital, LMU Munich, Munich, Germany
2 Centre for Brain Research, Indian Institute of Science, Bangalore, India
3 Stroke Research Group, Division of Clinical Neurosciences, University of Cambridge, UK
4 INSERM U1219 Bordeaux Population Health Research Center, Bordeaux, France
5 University of Bordeaux, Bordeaux, France
6 Laboratory for Statistical Analysis, RIKEN Center for Integrative Medical Sciences, Yokohama, Japan
7 Department of Statistical Genetics, Osaka University Graduate School of Medicine, Osaka, Japan
8 Laboratory of Statistical Immunology, Immunology Frontier Research Center (WPI-IFReC), Osaka University, Suita, Japan.
9 Department of Neurology, Massachusetts General Hospital, Harvard Medical School, Boston, MA, USA
10 Laboratory of Experimental Cardiology, Division of Heart and Lungs, University Medical Center Utrecht, University of Utrecht, Utrecht,Netherlands
11 deCODE genetics/AMGEN inc, Reykjavik, Iceland
12 Center for Genomic Medicine, Massachusetts General Hospital (MGH), Boston, MA, USA
13 J. Philip Kistler Stroke Research Center, Department of Neurology, MGH, Boston, MA, USA
14 Program in Medical and Population Genetics, Broad Institute, Cambridge, MA, USA
15 Population Health Research Institute, McMaster University, Hamilton, Canada
16 Department of Epidemiology, Erasmus University Medical Center, Rotterdam, Netherlands
17 Department of Radiology and Nuclear Medicine, Erasmus University Medical Center, Rotterdam, Netherlands
18 Department of Medicine and Clinical Science, Graduate School of Medical Sciences, Kyushu University, Fukuoka, Japan
19 Department of Clinical Sciences, Lund University, Malmö, Sweden
20 Univ. Lille, Inserm, Institut Pasteur de Lille, LabEx DISTALZ-UMR1167, Risk factors and molecular determinants of aging-related diseases, F-59000 Lille, France
21 Centre Hosp. Univ Lille, Epidemiology and Public Health Department, F-59000 Lille, France
22 AA Martinos Center for Biomedical Imaging, Department of Radiology, Massachusetts General Hospital, Harvard Medical School, Boston, MA, USA
23 Cardiovascular Health Research Unit, Departments of Biostatistics and Medicine, University of Washington, Seattle, WA, USA
24 Division of Neurology, Faculty of Medicine, Brain Research Center, University of British Columbia, Vancouver, Canada
25 School of Life Science, University of Lincoln, Lincoln, UK
26 Department of Cerebrovascular Diseases, Fondazione IRCCS Istituto Neurologico "Carlo Besta", Milano, Italy
27 Department of Neurology, Mayo Clinic Rochester, Rochester, MN, USA
28 MRC/BHF Cardiovascular Epidemiology Unit, Department of Public Health and Primary Care, University of Cambridge, Cambridge, UK
29 The National Institute for Health Research Blood and Transplant Research Unit in Donor Health and Genomics, University of Cambridge, UK
30 Neurovascular Research Laboratory, Vall d'Hebron Institut of Research, Neurology and Medicine Departments-Universitat Autònoma de Barcelona, Vall d'Hebrón Hospital, Barcelona, Spain
31 Stroke Pharmacogenomics and Genetics, Fundacio Docència i Recerca MutuaTerrassa, Terrassa, Spain
32 Children's Research Institute, Children's National Medical Center, Washington, DC, USA
33 Center for Translational Science, George Washington University, Washington, DC, USA
34 Division of Preventive Medicine, Brigham and Women's Hospital, Boston, MA, USA
35 Harvard Medical School, Boston, MA, USA
36 Center for Public Health Genomics, Department of Public Health Sciences, University of Virginia, Charlottesville, VA, USA

37 Department of Neurology, University of Maryland School of Medicine and Baltimore VAMC, Baltimore, MD, USA
38 Departments of Medicine, Pediatrics and Population Health Science, University of Mississippi Medical Center, Jackson, MS, USA
39 Institute of Cardiovascular Research, Royal Holloway University of London, UK & Ashford and St Peters Hospital, Surrey UK
40 Department of Psychiatry,The Hope Center Program on Protein Aggregation and Neurodegeneration (HPAN),Washington University, School of Medicine, St. Louis, MO, USA
41 Department of Developmental Biology, Washington University School of Medicine, St. Louis, MO, USA
42 NIHR Blood and Transplant Research Unit in Donor Health and Genomics, Department of Public Health and Primary Care, University of Cambridge, Cambridge, UK
43 Wellcome Trust Sanger Institute, Wellcome Trust Genome Campus, Hinxton, Cambridge, UK
44 British Heart Foundation, Cambridge Centre of Excellence, Department of Medicine, University of Cambridge, Cambridge, UK
45 Department of Medical Genetics, University Medical Center Utrecht, Utrecht, Netherlands
46 Department of Epidemiology, Julius Center for Health Sciences and Primary Care, University Medical Center Utrecht, Utrecht, Netherlands
47 Boston University School of Public Health, Boston, MA, USA
48 Framingham Heart Study, Framingham, MA, USA
49 Department of Immunology, Genetics and Pathology and Science for Life Laboratory, Uppsala University, Uppsala, Sweden
50 Department of Genetics, University of North Carolina, Chapel Hill, NC, USA
51 Department of Neurology and Stroke Center, Basel University Hospital, Switzerland
52 Neurorehabilitation Unit, University and University Center for Medicine of Aging and Rehabilitation Basel, Felix Platter Hospital, Basel, Switzerland
53 Department of Neurology, Yale University School of Medicine, New Haven, CT, USA
54 Program in Medical and Population Genetics, The Broad Institute of Harvard and MIT, Cambridge, MA, USA
55 Department of Neurology, Johns Hopkins University School of Medicine, Baltimore, MD, USA
56 Neuroscience Institute, SF Medical Center, Trenton, NJ, USA
57 Icelandic Heart Association Research Institute, Kopavogur, Iceland
58 University of Iceland, Faculty of Medicine, Reykjavik, Iceland
59 Department of Medical Sciences, Molecular Epidemiology and Science for Life Laboratory, Uppsala University, Uppsala, Sweden
60 Division of Public Health Sciences, Fred Hutchinson Cancer Research Center, Seattle, WA, USA
61 Laboratory of Epidemiology and Population Science, National Institute on Aging, National Institutes of Health, Bethesda, MD, USA
62 Department of Neurology, Leeds General Infirmary, Leeds Teaching Hospitals NHS Trust, Leeds, UK
63 National Institute for Health and Welfare, Helsinki, Finland
64 FIMM - Institute for Molecular Medicine Finland, Helsinki, Finland
65 Department of Epidemiology, University of Washington, Seattle, WA, USA
66 Public Health Stream, Hunter Medical Research Institute, New Lambton, Australia
67 Faculty of Health and Medicine, University of Newcastle, Newcastle, Australia
68 School of Public Health, University of Alabama at Birmingham, Birmingham, AL, USA
69 Department of Biostatistical Sciences, Wake Forest School of Medicine, Winston-Salem, NC, USA
70 Aflac Cancer and Blood Disorder Center, Department of Pediatrics, Emory University School of Medicine, Atlanta, GA, USA
71 Department of Medicine, Division of Cardiovascular Medicine, Stanford University School of Medicine, CA, USA
72 Department of Medical Sciences, Molecular Epidemiology and Science for Life Laboratory, Uppsala University, Uppsala, Sweden
73 Epidemiology, School of Public Health, University of Alabama at Birmingham, USA
74 Brown Foundation Institute of Molecular Medicine, University of Texas Health Science Center at Houston, Houston, TX, USA

75 Neurovascular Research Group (NEUVAS), Neurology Department, Institut Hospital del Mar d'Investigació Mèdica, Universitat Autònoma de Barcelona, Barcelona, Spain
76 Department of Pharmacotherapy and Translational Research and Center for Pharmacogenomics, University of Florida, College of Pharmacy, Gainesville, FL, USA
77 Division of Cardiovascular Medicine, College of Medicine, University of Florida, Gainesville, FL, USA
78 Department of Cardiology, Leiden University Medical Center, Leiden, the Netherlands
79 Program in Bioinformatics and Integrative Genomics, Harvard Medical School, Boston, MA, USA
80 Department of Biology, East Carolina University, Greenville, NC, USA
81 Center for Health Disparities, East Carolina University, Greenville, NC, USA
82 University of Cincinnati College of Medicine, Cincinnati, OH, USA
83 RIKEN Center for Integrative Medical Sciences, Yokohama, Japan
84 Department of Medicine, University of Colorado Denver, Anschutz Medical Campus, Aurora, CO, USA
85 Center for Public Health Genomics and Department of Biostatistical Sciences, Wake Forest School of Medicine, Winston-Salem, NC, USA
86 MRC Epidemiology Unit, University of Cambridge School of Clinical Medicine, Institute of Metabolic Science, Cambridge Biomedical Campus, Cambridge, UK
87 Intramural Research Program, National Institute on Aging, National Institutes of Health, Bethesda, MD, USA
88 Department of Neurology, Radiology, and Biomedical Engineering, Washington University School of Medicine, St. Louis, MO, USA
89 KU Leuven – University of Leuven, Department of Neurosciences, Experimental Neurology, Leuven, Belgium
90 VIB Center for Brain & Disease Research, University Hospitals Leuven, Department of Neurology, Leuven, Belgium
91 Univ.-Lille, INSERM U 1171. CHU Lille. Lille, France
92 Department of Medical and Molecular Genetics, King's College London, London, UK
93 SGDP Centre, Institute of Psychiatry, Psychology & Neuroscience, King's College London, London, UK
94 Northern Institute for Cancer Research, Paul O'Gorman Building, Newcastle University, Newcastle, UK
95 Department of Clinical Sciences Lund, Neurology, Lund University, Lund, Sweden
96 Department of Neurology and Rehabilitation Medicine, Skåne University Hospital, Lund, Sweden
97 Bioinformatics Core Facility, University of Gothenburg, Gothenburg, Sweden
98 Department of Medical Epidemiology and Biostatistics, Karolinska Institutet, Stockholm, Sweden
99 University of Technology Sydney, Faculty of Health, Ultimo, Australia
100 Department of Medicine, University of Maryland School of Medicine, MD, USA
101 Department of Neurology, Mayo Clinic, Jacksonville, FL, USA
102 Geriatrics Research and Education Clinical Center, Baltimore Veterans Administration Medical Center, Baltimore, MD, USA
103 Division of Geriatrics, School of Medicine, University of Mississippi Medical Center, Jackson, MS, USA
104 Memory Impairment and Neurodegenerative Dementia Center, University of Mississippi Medical Center, Jackson, MS, USA
105 Laboratory of Neurogenetics, National Institute on Aging, National institutes of Health, Bethesda, MD, USA
106 Data Tecnica International, Glen Echo MD, USA
107 Department of Epidemiology and Public Health, Graduate School of Medical Sciences, Kyushu University, Fukuoka, Japan
108 Clinical Research Facility, Department of Medicine, NUI Galway, Galway, Ireland
109 Cardiovascular Health Research Unit, Department of Medicine, University of Washington, Seattle, WA, USA
110 Department of Epidemiology, University of Washington, Seattle, WA
111 Department of Health Services, University of Washington, Seattle, WA, USA

112 Kaiser Permanente Washington Health Research Institute, Seattle, WA, USA
113 Brain Center Rudolf Magnus, Department of Neurology, University Medical Center Utrecht, Utrecht, The Netherlands
114 Usher Institute of Population Health Sciences and Informatics, University of Edinburgh, Edinburgh, UK
115 Centre for Clinical Brain Sciences, University of Edinburgh, Edinburgh, UK
116 Fred Hutchinson Cancer Research Center, University of Washington, Seattle, WA, USA
117 Department of Medicine, Brigham and Women's Hospital, Boston, MA, USA
118 Department of Biostatistics, University of Washington, Seattle, WA, USA
119 Nuffield Department of Clinical Neurosciences, University of Oxford, UK
120 Institute for Translational Genomics and Population Sciences, Los Angeles Biomedical Research Institute at Harbor-UCLA Medical Center, Torrance, CA, USA
121 Division of Genomic Outcomes, Department of Pediatrics, Harbor-UCLA Medical Center, Torrance, CA, USA
122 Department of Neurology, Miller School of Medicine, University of Miami, Miami, FL, USA
123 Department of Allergy and Rheumatology, Graduate School of Medicine, the University of Tokyo, Tokyo, Japan
124 Center for Public Health Genomics, University of Virginia, Charlottesville, VA, USA
125 Department of Pediatrics, College of Medicine, University of Oklahoma Health Sciences Center, Oklahoma City, OK, USA
126 Department of Neurology, Medical University of Graz, Graz, Austria
127 University Medicine Greifswald, Institute for Community Medicine, SHIP-KEF, Greifswald, Germany
128 University Medicine Greifswald, Department of Neurology, Greifswald, Germany
129 Department of Neurology, Jagiellonian University, Krakow, Poland
130 Department of Neurology, Justus Liebig University, Giessen, Germany
131 Department of Clinical Neurosciences/Neurology, Institute of Neuroscience and Physiology, Sahlgrenska Academy at University of Gothenburg, Gothenburg, Sweden
132 Sahlgrenska University Hospital, Gothenburg, Sweden
133 Stroke Division, Florey Institute of Neuroscience and Mental Health, University of Melbourne, Heidelberg, Australia
134 Austin Health, Department of Neurology, Heidelberg, Australia
135 Department of Internal Medicine, Section Gerontology and Geriatrics, Leiden University Medical Center, Leiden, the Netherlands
136 INSERM U1219, Bordeaux, France
137 Department of Public Health, Bordeaux University Hospital, Bordeaux, France
138 Genetic Epidemiology Unit, Department of Epidemiology, Erasmus University Medical Center Rotterdam, Netherlands
139 Center for Medical Systems Biology, Leiden, Netherlands
140 School of Medicine, Dentistry and Nursing at the University of Glasgow, Glasgow, UK
141 Department of Epidemiology and Population Health, Albert Einstein College of Medicine, NY, USA
142 Department of Physiology and Biophysics, University of Mississippi Medical Center, Jackson, MS, USA
143 A full list of members and affiliations appears in the Supplementary Note
144 Department of Human Genetics, McGill University, Montreal, Canada
145 Department of Pathophysiology, Institute of Biomedicine and Translation Medicine, University of Tartu, Tartu, Estonia
146 Department of Cardiac Surgery, Tartu University Hospital, Tartu, Estonia
147 Clinical Gene Networks AB,Stockholm, Sweden
148 Department of Genetics and Genomic Sciences, The Icahn Institute for Genomics and Multiscale Biology Icahn School of Medicine at Mount Sinai, New York, NY , USA
149 Department of Pathophysiology, Institute of Biomedicine and Translation Medicine, University of Tartu, Biomeedikum, Tartu, Estonia
150 Integrated Cardio Metabolic Centre, Department of Medicine, Karolinska Institutet, Karolinska Universitetssjukhuset, Huddinge, Sweden.
151 Clinical Gene Networks AB, Stockholm, Sweden

152 Sorbonne Universités, UPMC Univ. Paris 06, INSERM, UMR_S 1166, Team Genomics & Pathophysiology of Cardiovascular Diseases, Paris, France
153 ICAN Institute for Cardiometabolism and Nutrition, Paris, France
154 Department of Biomedical Engineering, University of Virginia, Charlottesville, VA, USA
155 Group Health Research Institute, Group Health Cooperative, Seattle, WA, USA
156 Seattle Epidemiologic Research and Information Center, VA Office of Research and Development, Seattle, WA, USA
157 Cardiovascular Research Center, Massachusetts General Hospital, Boston, MA, USA
158 Department of Medical Research, Bærum Hospital, Vestre Viken Hospital Trust, Gjettum, Norway
159 Saw Swee Hock School of Public Health, National University of Singapore and National University Health System, Singapore
160 National Heart and Lung Institute, Imperial College London, London, UK
161 Department of Gene Diagnostics and Therapeutics, Research Institute, National Center for Global Health and Medicine, Tokyo, Japan
162 Department of Epidemiology, Tulane University School of Public Health and Tropical Medicine, New Orleans, LA, USA
163 Department of Cardiology,University Medical Center Groningen, University of Groningen, Netherlands
164 MRC-PHE Centre for Environment and Health, School of Public Health, Department of Epidemiology and Biostatistics, Imperial College London, London, UK
165 Department of Epidemiology and Biostatistics, Imperial College London, London, UK
166 Department of Cardiology, Ealing Hospital NHS Trust, Southall, UK
167 National Heart, Lung and Blood Research Institute, Division of Intramural Research, Population Sciences Branch, Framingham, MA, USA
168 A full list of members and affiliations appears at the end of the manuscript
169 Department of Phamaceutical Sciences, Collge of Pharmacy, University of Oklahoma Health Sciences Center, Oklahoma City, OK, USA
170 Oklahoma Center for Neuroscience, Oklahoma City, OK, USA
171 Department of Pathology and Genetics, Institute of Biomedicine, The Sahlgrenska Academy at University of Gothenburg, Gothenburg, Sweden
172 Department of Neurology, Helsinki University Hospital, Helsinki, Finland
173 Clinical Neurosciences, Neurology, University of Helsinki, Helsinki, Finland
174 Department of Neurology, University of Washington, Seattle, WA, USA
175 Albrecht Kossel Institute, University Clinic of Rostock, Rostock, Germany
176 Clinical Trial Service Unit and Epidemiological Studies Unit, Nuffield Department of Population Health, University of Oxford, Oxford, UK
177 Department of Genetics, Perelman School of Medicine, University of Pennsylvania, PA, USA
178 Faculty of Medicine, University of Iceland, Reykjavik, Iceland
179 Departments of Neurology and Public Health Sciences, University of Virginia School of Medicine, Charlottesville, VA, USA
180 Department of Neurology, Boston University School of Medicine, Boston, MA, USA
181 Human Genetics Center, University of Texas Health Science Center at Houston, Houston, TX, USA
182 Center for Genomic Medicine, Kyoto University Graduate School of Medicine, Kyoto, Japan
183 Munich Cluster for Systems Neurology (SyNergy), Munich, Germany
184 German Center for Neurodegenerative Diseases (DZNE), Munich, Germany
185 Boston University School of Medicine, Boston, MA, USA
186 University of Kentucky College of Public Health, Lexington, KY, USA
187 University of Newcastle and Hunter Medical Research Institute, New Lambton, Australia
188 Univ. Montpellier, Inserm, U1061, Montpellier, France
189 Centre for Research in Environmental Epidemiology, Barcelona, Spain
190 Department of Neurology, Università degli Studi di Perugia, Umbria, Italy
191 Department of Medicine, University of Maryland School of Medicine, Baltimore, MD, USA
192 Broad Institute, Cambridge, MA, USA
193 Univ. Bordeaux, Inserm, Bordeaux Population Health Research Center, UMR 1219, Bordeaux, France
194 Bordeaux University Hospital, Department of Neurology, Memory Clinic, Bordeaux, France

195 Neurovascular Research Laboratory. Vall d'Hebron Institut of Research, Neurology and Medicine Departments-Universitat Autònoma de Barcelona. Vall d'Hebrón Hospital, Barcelona, Spain
196 University Medicine Greifswald, Department of Internal Medicine B, Greifswald, Germany
197 DZHK, Greifswald, Germany
198 Robertson Center for Biostatistics, University of Glasgow, Glasgow, UK
199 Hero DMC Heart Institute, Dayanand Medical College & Hospital, Ludhiana, India
200 Atherosclerosis Research Unit, Department of Medicine Solna, Karolinska Institutet, Stockholm, Sweden
201 Karolinska Institutet, Stockholm, Sweden
202 Division of Emergency Medicine, and Department of Neurology, Washington University School of Medicine, St. Louis, MO, USA
203 Tohoku Medical Megabank Organization, Sendai, Japan
204 Department of Psychiatry, Washington University School of Medicine, St. Louis, MO, USA
205 Department of Public Health and Caring Sciences / Geriatrics, Uppsala University, Uppsala, Sweden
206 Epidemiology and Prevention Group, Center for Public Health Sciences, National Cancer Center, Tokyo, Japan
207 Department of Internal Medicine and the Center for Clinical and Translational Science, The Ohio State University, Columbus, OH, USA
208 Institute of Neuroscience and Physiology, the Sahlgrenska Academy at University of Gothenburg, Goteborg, Sweden
209 Department of Basic and Clinical Neurosciences, King's College London, London, UK
210 Department of Health Care Administration and Management, Graduate School of Medical Sciences, Kyushu University, Japan
211 Department of Medicine and Clinical Science, Graduate School of Medical Sciences, Kyushu University, Japan
212 Landspitali National University Hospital, Departments of Neurology & Radiology, Reykjavik, Iceland
213 Department of Neurology, Heidelberg University Hospital, Germany
214 Department of Neurology, Erasmus University Medical Center
215 Hospital Universitari Mutua Terrassa, Terrassa (Barcelona), Spain
216 Albert Einstein College of Medicine, Montefiore Medical Center, New York, NY, USA
217 John Hunter Hospital, Hunter Medical Research Institute and University of Newcastle, Newcastle, NSW, Australia
218 Centre for Prevention of Stroke and Dementia, Nuffield Department of Clinical Neurosciences, University of Oxford, UK
219 Department of Medical Sciences, Uppsala University, Uppsala, Sweden
220 Genetic and Genomic Epidemiology Unit, Wellcome Trust Centre for Human Genetics, University of Oxford, Oxford, UK
221 The Wellcome Trust Centre for Human Genetics, Oxford, UK
222 Beth Israel Deaconess Medical Center, Boston, MA, USA
223 Wake Forest School of Medicine, Wake Forest, NC, USA
224 Department of Neurology, University of Pittsburgh, Pittsburgh, PA, USA
225 BioBank Japan, Laboratory of Clinical Sequencing, Department of Computational biology and medical Sciences, Graduate school of Frontier Sciences, The University of Tokyo, Tokyo, Japan
226 Neurovascular Research Laboratory, Vall d'Hebron Institut of Research, Neurology and Medicine Departments-Universitat Autònoma de Barcelona. Vall d'Hebrón Hospital, Barcelona, Spain
227 Department of Biostatistics, University of Liverpool, Liverpool, UK
228 Wellcome Trust Centre for Human Genetics, University of Oxford, Oxford, UK
229 Institute of Genetic Epidemiology, Helmholtz Zentrum München - German Research Center for Environmental Health, Neuherberg, Germany
230 Department of Medicine I, Ludwig-Maximilians-Universität, Munich, Germany
231 DZHK (German Centre for Cardiovascular Research), partner site Munich Heart Alliance, Munich, Germany
232 Department of Cerebrovascular Diseases, Fondazione IRCCS Istituto Neurologico "Carlo Besta", Milano, Italy

233 Karolinska Institutet, MEB, Stockholm, Sweden
234 University of Tartu, Estonian Genome Center, Tartu, Estonia, Tartu, Estonia
235 Department of Clinical and Experimental Sciences, Neurology Clinic, University of Brescia, Italy
236 Translational Genomics Unit, Department of Oncology, IRCCS Istituto di Ricerche Farmacologiche Mario Negri, Milano, Italy
237 Department of Genetics, Microbiology and Statistics, University of Barcelona, Barcelona, Spain
238 Psychiatric Genetics Unit, Group of Psychiatry, Mental Health and Addictions, Vall d'Hebron Research Institute (VHIR), Universitat Autònoma de Barcelona, Biomedical Network Research Centre on Mental Health (CIBERSAM), Barcelona, Spain
239 Department of Neurology, IMIM-Hospital del Mar, and Universitat Autònoma de Barcelona, Spain
240 IMIM (Hospital del Mar Medical Research Institute), Barcelona, Spain
241 National Institute for Health Research Comprehensive Biomedical Research Centre, Guy's & St. Thomas' NHS Foundation Trust and King's College London, London, UK
242 Division of Health and Social Care Research, King's College London, London, UK
243 FIMM-Institute for Molecular Medicine Finland, Helsinki, Finland
244 THL-National Institute for Health and Welfare, Helsinki, Finland
245 Iwate Tohoku Medical Megabank Organization, Iwate Medical University, Iwate, Japan
246 BHF Glasgow Cardiovascular Research Centre, Faculty of Medicine, Glasgow, UK
247 deCODE Genetics/Amgen, Inc., Reykjavik, Iceland
248 Icelandic Heart Association, Reykjavik, Iceland
249 Institute of Biomedicine, the Sahlgrenska Academy at University of Gothenburg, Goteborg, Sweden
250 Department of Epidemiology, University of Maryland School of Medicine, Baltimore, MD, USA
251 Institute of Cardiovascular and Medical Sciences, Faculty of Medicine, University of Glasgow, Glasgow, UK
252 Chair of Genetic Epidemiology, IBE, Faculty of Medicine, LMU Munich, Germany
253 Division of Epidemiology and Prevention, Aichi Cancer Center Research Institute, Nagoya, Japan
254 Department of Epidemiology, Nagoya University Graduate School of Medicine, Nagoya, Japan
255 University Medicine Greifswald, Institute for Community Medicine, SHIP-KEF, Greifswald, Germany
256 Department of Neurology, Caen University Hospital, Caen, France
257 University of Caen Normandy, Caen, France
258 Department of Internal Medicine, Erasmus University Medical Center, Rotterdam, Netherlands
259 Landspitali University Hospital, Reykjavik, Iceland
260 Survey Research Center, University of Michigan, Ann Arbor, MI, USA
261 University of Virginia Department of Neurology, Charlottesville, VA, USA

REFERENCES

1. Miller, C.L. *et al.* Integrative functional genomics identifies regulatory mechanisms at coronary artery disease loci. *Nat Commun* **7**, 12092 (2016).
2. Kalluri, A.S. *et al.* Single-Cell Analysis of the Normal Mouse Aorta Reveals Functionally Distinct Endothelial Cell Populations. *Circulation* **140**, 147-163 (2019).
