# Supplementary material for: Genetic investigation of fibromuscular dysplasia identifies risk loci and shared genetics with common cardiovascular diseases
Source: Nat Commun. 2021 Oct 15;12:6031. doi: 10.1038/s41467-021-26174-2 (PMC8521585; doi:10.1038/s41467-021-26174-2)
Supplement: Supplementary file 3 — Description of Additional Supplementary Files [file 41467_2021_26174_MOESM3_ESM.pdf]

Description of Additional Supplementary Files

File Name: Supplementary Data 1
Description: **Top associated SNPs with FMD in the meta-analysis.**
We display betas and P-values estimates for the association with FMD, for lead SNPs at each locus (P-value < 0.0001), obtained by LD clumping using PLINK package and LD data from the European panel of the 1000 Genomes study (Phase 3).
CHROM: chromosome, EAF: effect allele frequency, BETA: effect size, SE, standard error of BETA

File Name: Supplementary Data 2
Description: **Lookup of FMD association among genome-wide associated systolic blood pressure variants.**
We display betas and P-values estimates for the association with SBP and mFMD, for lead SNPs at each locus (FDR < 0.05), obtained by LD clumping using PLINK package and LD data from the European panel of the 1000 Genomes study (Phase 3).
CHROM: chromosome, EAF: effect allele frequency, BETA: effect size, SE: standard error of BETA, P.adj (bonf): Bonferroni adjusted P-value based on the testing of 821 SBP associated variants, FDR: false discovery rate.
