# Supplementary material for: Genetic investigation of fibromuscular dysplasia identifies risk loci and shared genetics with common cardiovascular diseases
Source: Nat Commun. 2021 Oct 15;12:6031. doi: 10.1038/s41467-021-26174-2 (PMC8521585; doi:10.1038/s41467-021-26174-2)
Supplement: Supplementary file 6 — Reporting Summary [file 41467_2021_26174_MOESM6_ESM.pdf]

nature portfolio

Corresponding author(s): Nabila Bouatia-Naji, Santhi K. Ganesh

Last updated by author(s): Aug 25, 2021

Reporting Summary

Nature Portfolio wishes to improve the reproducibility of the work that we publish. This form provides structure for consistency and transparency in reporting. For further information on Nature Portfolio policies, see our Editorial Policies and the Editorial Policy Checklist.

Statistics

For all statistical analyses, confirm that the following items are present in the figure legend, table legend, main text, or Methods section.

n/a	Confirmed	
☐	☒	The exact sample size (*n*) for each experimental group/condition, given as a discrete number and unit of measurement
☒	☐	A statement on whether measurements were taken from distinct samples or whether the same sample was measured repeatedly
☐	☒	The statistical test(s) used AND whether they are one- or two-sided<br>*Only common tests should be described solely by name; describe more complex techniques in the Methods section.*
☐	☒	A description of all covariates tested
☐	☒	A description of any assumptions or corrections, such as tests of normality and adjustment for multiple comparisons
☐	☒	A full description of the statistical parameters including central tendency (e.g. means) or other basic estimates (e.g. regression coefficient) AND variation (e.g. standard deviation) or associated estimates of uncertainty (e.g. confidence intervals)
☐	☒	For null hypothesis testing, the test statistic (e.g. *F*, *t*, *r*) with confidence intervals, effect sizes, degrees of freedom and *P* value noted<br>*Give P values as exact values whenever suitable.*
☐	☒	For Bayesian analysis, information on the choice of priors and Markov chain Monte Carlo settings
☒	☐	For hierarchical and complex designs, identification of the appropriate level for tests and full reporting of outcomes
☐	☒	Estimates of effect sizes (e.g. Cohen's *d*, Pearson's *r*), indicating how they were calculated

Our web collection on statistics for biologists contains articles on many of the points above.

Software and code

Policy information about availability of computer code

Data collection	(1) We queried GTEx database (v8 release) with rsID of lead variants at FMD loci<br>(2) Gene expression models for TWAS were precomputed from GTEx data (v7 release) and were provided by the authors of FUSION package and were retrieved on June 7th 2021 from [http://gusevlab.org/projects/fusion/].<br>(3) GWAS catalog database was queried on 2020-08-05
Data analysis	(1) GWAS was conducted in each study under an additive genetic model using PLINK v2.0.<br>(2) Association results were combined using an inverse variance weighted fixed-effects meta-analysis in METAL software (2011-03-25 release).<br>(3) LocusZoom (http://locuszoom.org/) was used to provide regional visualization of results.<br>(4) Gene based association was conducted using the MAGMA tool (v1.08), implemented in the FUMA platform (v1.3.6a)<br>(5) TWAS was performed using FUSION R/python package. FUSION R/python package source code was retrieved on June 7th 2021 from https://github.com/gusevlab/fusion_twas.<br>(6) Statistical analyses and plotting were performed using R (3.6.1), through RStudio interface software (v1.2.335). Following packages were used: MatrixEQTL_v.2.1.1, LDlinkR_1.0.2, ggrepel_0.8.2, locuscomparer_1.0.0, coloc_3.2-1, dplyr_1.0.2, tidyr_1.1.1, RColorBrewer_1.1-2, ggplot2_3.3.3.<br>(7) Sequencing reads were demultiplexed using bcl2fastq2-2.18.12.<br>(8) Adapter sequences were trimmed using CutAdapt v1.15.<br>(9) Analyse of ATAC-Seq data (steps 10-17) was performed on the Galaxy webserver (https://usegalaxy.org/)<br>(10) Reads were mapped on GRCh38 (hg38) genome using Bowtie2 v2.3.4.3.<br>(11) Aligned reads were filtered using BAM filter v0.5.9<br>(12) ATAC peaks were called using MACS2 callpeak v2.1.1.20160309.6<br>(13) Binary read density files (bigwig) were created using bamCoverage v3.3.0.0.0<br>(14) A common list of enriched regions was generated using bedtools multiple intersect (Galaxy Version 2.29.0)

(15) Average read coverage on these regions was computed using deepTools multiBamsummary (Galaxy Version 3.3.2.0.0)
(16) deepTools plotCoverage and plotPCA functions (Galaxy Version 3.3.2.0.0) were used to calculate Spearman correlation between samples and Principal Component Analysis, respectively.
(17) We used Diffbind (Galaxy Version 2.10.0) to detect differentially accessible regions between Artery and SMC and Artery and EC samples.

(18) Global peak annotation was performed using ChIPSeeker v1.22.0.
(19) clusterProfiler v3.14.0 was used to annotate genes at proximity (≤10kb, 1425 genes) of ATAC-Seq peaks and identify enriched gene ontology terms.
(20) Identified GOBP terms were clustered using REVIGO webserver (http://revigo.irb.hr/).
(21) We used DAVID webserver (v6.7, https://david.ncifcrf.gov/) to perform functional annotation clustering.
(22) We computed the overlap of variant with open chromatin regions histone-ChIP peaks using bedtools (v2.29.0) annotate function.
(23) We used Integrated Genome Browser (IGB, v9.1.4) to visualize read density profiles and peak positions in the context of human genome
(24) Analysis of SNP enrichment among ATAC-Seq peaks was performed using GREGOR perl package (v1.4.0).

(25) For transcriptomic analysis, sequencing reads (fastq files) were mapped to GRCh38 human reference genome using STAR aligner (version 3.6.0c)
(26) Differential gene expression was analyzed by R (3.5.1) package sva (sva_3.34.0)

(27) We used ldsc package (v1.0.1, https://github.com/bulik/ldsc/) to assess the SNP-based heritability (h2) of FMD and to estimate genetic correlation between FMD and other diseases and traits.
(28) We conditioned FMD association on systolic blood pressure genetic association using multi-trait-based conditional and joint analysis (mtCOJO) tool from GCTA pipeline (v1.93.2beta).

For manuscripts utilizing custom algorithms or software that are central to the research but not yet described in published literature, software must be made available to editors and reviewers. We strongly encourage code deposition in a community repository (e.g. GitHub). See the Nature Portfolio guidelines for submitting code & software for further information.

Data

Policy information about availability of data

All manuscripts must include a data availability statement. This statement should provide the following information, where applicable:

- Accession codes, unique identifiers, or web links for publicly available datasets
- A description of any restrictions on data availability
- For clinical datasets or third party data, please ensure that the statement adheres to our policy

(1)The GWAS data generated in this study (Figure 1, etc.) have been deposited in the GWAS catalog database under accession code GCST90026612.
(2) Summary statistics data used for genetic correlation were acquired from the respective consortia: Hypertension (HTN) [http://www.nealelab.is/uk-biobank], Systolic Blood Pressure (SBP) [ftp://ftp.ebi.ac.uk/pub/databases/gwas/summary_statistics/EvangelouE_30224653_GCST006624], Diastolic Blood Pressure (DBP) [ftp://ftp.ebi.ac.uk/pub/databases/gwas/summary_statistics/EvangelouE_30224653_GCST006630], Pulse Pressure (PP) [ftp://ftp.ebi.ac.uk/pub/databases/gwas/summary_statistics/EvangelouE_30224653_GCST006629], Coronary Artery Disease (CAD) [http://www.cardiogramplusc4d.org/data-downloads/], Myocardial Infarction (MI) [http://www.cardiogramplusc4d.org/data-downloads/], Migraine [http://www.nealelab.is/uk-biobank], Any stroke (AS) [https://www.megastroke.org/], Any Ischemic Stroke (AIS) [https://www.megastroke.org/], Large Artery Stroke (LAS) [https://www.megastroke.org/], Cardioembolic Stroke (CES) [https://www.megastroke.org/], Small Vessel Stroke (SVS) [https://www.megastroke.org/], Intracranial Aneurysm (IA) [DOI: 10.1038/s41588-020-00725-7], Subarachnoid Haemorrhage (SAH) [DOI: 10.1038/s41588-020-00725-7], unruptured Intracranial Aneurysm (uIA) [DOI: 10.1038/s41588-020-00725-7], Cervical Artery Dissection (CeAD) [DOI: 10.1038/ng.3154], HDL [http://www.nealelab.is/uk-biobank], LDL [http://www.nealelab.is/uk-biobank], Total cholesterol (TC) [http://www.nealelab.is/uk-biobank], Trygliceryde (TG) [http://www.nealelab.is/uk-biobank], Apolipoprotein A (ApoA) [http://www.nealelab.is/uk-biobank], Apolipoprotein B (ApoB) [http://www.nealelab.is/uk-biobank], Creatinine [http://www.nealelab.is/uk-biobank], CystatinC [http://www.nealelab.is/uk-biobank], eGFR creatinine [https://ckdgen.imbi.uni-freiburg.de], eGFR cystatinC [https://ckdgen.imbi.uni-freiburg.de], UACR [https://ckdgen.imbi.uni-freiburg.de], BUN [https://ckdgen.imbi.uni-freiburg.de], Urate [http://www.nealelab.is/uk-biobank], Urea [http://www.nealelab.is/uk-biobank], CRP [http://www.nealelab.is/uk-biobank]
(3) The ATAC-Seq sequencing data generated in this study (Figure S5, Figure3, Table 3) are publicly available on the Sequence Read Archive under accession code PRJNA690001 [https://www.ncbi.nlm.nih.gov/bioproject/690001].
(4) Sequencing reads for ATAC-Seq experiments on coronary arteries (Figure S5, Figure3, Table 3) were retrieved from the Sequence Read Archive (SRR2378591, SRR2378592, SRR2378593)
(5) Peak files (Table 3) were retrieved from the following experiments in ENCODE database (ENCFF874DSY, ENCFF595RQJ, ENCFF208DZK, ENCFF722XLA, ENCFF776MXU, ENCFF121HDP, ENCFF028BAF, ENCFF030AUO, ENCFF003NBP, ENCFF967DLF, ENCFF134QRV, ENCFF020COG, ENCFF316LHC, ENCFF914DAA, ENCFF974YFN, ENCFF980YJQ, ENCFF256LHH, ENCFF194TWC, ENCFF340GNE, ENCFF080KQQ, ENCFF831ZKB, ENCFF821NNE, ENCFF651FRA, ENCFF633GZP, ENCFF154GIE, ENCFF682FCZ, ENCFF608ZGR, ENCFF682ELX, ENCFF647QUI, ENCFF313MVM, ENCFF701WDJ, ENCFF817XTD, ENCFF205QFR, ENCFF582FDJ, ENCFF946FND, ENCFF930ZZM, ENCFF593YQY)
(6) The following read density files were retrieved from ENCODE database for visualization (Figure 3) (ENCFF749KKG, ENCFF715CEI, ENCFF721SMI, ENCFF891TZE)
(7) Single-cell sequencing in aorta (Supplementary figure S6) was looked-up at Single cell portal [https://singlecell.broadinstitute.org/single_cell/study/SCP289/]
(8) Tibial artery expression dataset (Supplementary figure S7) was retrieved from GTEx consortium (v8) [https://storage.googleapis.com/gtex_analysis_v8/rna_seq_data/GTEx_Analysis_2017-06-05_v8_RNASeQCv1.1.9_gene_tpm.gct.gz]

Field-specific reporting

Please select the one below that is the best fit for your research. If you are not sure, read the appropriate sections before making your selection.

☒ Life sciences ☐ Behavioural & social sciences ☐ Ecological, evolutionary & environmental sciences

For a reference copy of the document with all sections, see nature.com/documents/nr-reporting-summary-flat.pdf

Life sciences study design

All studies must disclose on these points even when the disclosure is negative.

Sample size	(1) GWAS meta-analysis: Recruitment of FMD patients was the main limiting factor for sample size of GWAS case-control studies and by far represents the more thorough genetic investigation in FMD. In 5 out of 6 case-control studies, the number of controls was picked to have a minimum 2.5x ratio over FMD cases, in order to allow a robust measure of variant frequency, and maximum ratio of 10x the number of FMD cases, to avoid artifical inflation of effective sample size. Number of controls in DEFINE study was more limited due to extensive clinical characterization of control patients, which was compensated by a careful matching by sex and age of case and control populations prior to genetic analysis.<br><br>(2) ATAC-Seq : Three biologically independent primary cell lines were analyed for each of the sample groups (smooth muscle cells, endothelial cells, fibroblasts). This was chosen to have a representative set of open chromatin regions and as a minimal sample size to allow statistical testing (for Supplementary Figure S5d-f). The high level of clustering between samples (Supplementary Figure S5b-c) suggests that a higher sample size was not required.<br><br>(3) Fibroblasts eQTL: As for the genetic study, recruitment of FMD patients was the main limiting factor for sample size selection and additional samples were not available.
Data exclusions	(1) FMD lesions may present with heterogenous characteristics and are generally classified in two subtypes, multifocal (by far the most frequent) and focal. In two case-control studies (UM-MGI/CCF and DEFINE-FMD), FMD patients with focal lesions were excluded from recruitment. For consistency and following a reviewer's comment, FMD cases with focal or uncharacterized lesions were therefore excluded from all case-control studies and from the global meta-analysis. The number of focal FMD cases was too low to conduct a specific association study.<br>Non-European cases and controls were excluded based on PCA analysis, except in UM-MGI/CCF case control study where nine non-European cases were kept and matched for controls by PCs coordinated.<br><br>(2) No exclusion for other datasets.
Replication	(1) The 6 case-control studies presented in the meta-analysis are the only genetic studies on FMD to our knowledge, and further replications could not be led. Homogeneity of association between the 6 studies was confirmed at all identified loci.<br><br>(2) ATAC-Seq experiments were ran in triplicates and pooled for sequencing.
Randomization	(1) Individuals were allocated to patients and control groups through clinical recruitment and could not be randomized. To adjust for population covariates, genetic models were adjusted for population structure using the first five principal components, sex (except in the women-only analyses) and study specific genomic control.<br><br>(2) Randomization was not applicable to genomic experiments in the study (ATAC-Seq), as different treatments (cell culture protocols) were applied depending of the nature of samples.<br><br>(3) For FMD fibroblasts isolation and culture, investigators were dependent on fresh tissue collection that required immediate culture and analysis. Therefore samples were included based on date of collection and could not be randomized.
Blinding	(1) The investigators could not be blinded during data collection and analysis, as case and control cohorts originated from different protocols and/or required a thorough clinical investigation.<br><br>(2) Blinding was not applicable to genomic experiments in the study (ATAC-Seq), as different treatments (cell culture protocols) were applied depending of the nature of samples.<br><br>(3) FMD fibroblasts isolation and culture also involved the isolation of fibroblasts from control individuals within DEFINE-FMD protocol. Samples were identified by an inclusion number based on the rank of patient inclusion within DEFINE-FMD, which did not refer to FMD status of the patients. Therefore, investigators were blind to FMD status of samples during cell culture and RNA-sequencing.

Reporting for specific materials, systems and methods

We require information from authors about some types of materials, experimental systems and methods used in many studies. Here, indicate whether each material, system or method listed is relevant to your study. If you are not sure if a list item applies to your research, read the appropriate section before selecting a response.

Materials & experimental systems

n/a	Involved in the study
[x]	[ ] Antibodies
[ ]	[x] Eukaryotic cell lines
[x]	[ ] Palaeontology and archaeology
[x]	[ ] Animals and other organisms
[ ]	[x] Human research participants
[x]	[ ] Clinical data
[x]	[ ] Dual use research of concern

Methods

n/a	Involved in the study
[x]	[ ] ChIP-seq
[x]	[ ] Flow cytometry
[x]	[ ] MRI-based neuroimaging

Eukaryotic cell lines

Policy information about cell lines

Cell line source(s)

(1) Fibroblasts of the DEFINE-FMD study were derived from skin biopsy samples using standard explant techniques from 83 FMD patients participating to the genetic study.
(2) Primary cells used for ATAC-Seq were purchased from Cell Applications (San Diego, CA) except HDF (ATCC, Manassas, VA).

Authentication

Only early passages of primary cell lines were used and none of the cell lines were further authenticated.

Mycoplasma contamination

Purchased cell lines were mycoplasma tested by providers. Only early passages of primary cell lines were used and mycoplasma testing was not ran.

Commonly misidentified lines
(See ICLAC register)

None

Human research participants

Policy information about studies involving human research participants

Population characteristics

Quantitative traits are indicated as Mean±SD.

(1) ARCADIA, France, N: 431, N Women (%): 376 (87.2), Age (Inclusion): 55.68± 12.49, Age (FMD diagnosis): 53.50 ± 13.09, BMI (kg/m2):23.74 ± 4.28, N Hypertension (%): 344 (79.8)
(2) 3 Cities study, France, N: 1487, N Women (%): 876 (58.9), Age (Inclusion): 74.36 ± 5.5, BMI (kg/m2):NA, N Hypertension (%): 1171 (78.7)
(3) ARCADIA-POL, Poland, N: 107, N Women (%): 88 (82.2), Age (Inclusion): 47.82 ± 13.23, Age (FMD diagnosis): 45.35 ± 13.46, BMI (kg/m2):24.80 ± 4.01, N Hypertension (%): 91 (85%)
(4) WOBASZ II, Poland, N: 295, N Women (%): 227 (76.9), Age (Inclusion): 40.97 ± 11.05, BMI (kg/m2):26.37 ± 5.43, N Hypertension (%): 59
(5) FEIRI, Europe, N: 243, N Women (%): 215 (88.5), Age (Inclusion): NA, Age (FMD diagnosis): 50.60 ± 12.96, BMI (kg/m2):NA, N Hypertension (%): NA
(6) ASKLEPIOS, Europe, N: 615, N Women (%): 611 (99.3), Age (Inclusion): 45.67 ± 6.23, BMI (kg/m2):NA, N Hypertension (%): NA
(7) MayoVDB, USA, N: 116, N Women (%): 100 (86.2), Age (Inclusion): 58.78 ± 12.71, Age (FMD diagnosis): NA, BMI (kg/m2):26.87 ± 6.06, N Hypertension (%): 82 (70.7)
(8) MayoVDB, USA, N: 1141, N Women (%): 572 (50.1), Age (Inclusion): 64.98 ± 11.30, BMI (kg/m2):29.33 ± 6.02, N Hypertension (%): 680 (59.6)
(9) DEFINE-FMD, USA, N: 108, N Women (%): 108 (100.0), Age (Inclusion): 56.13 ± 10.00, Age (FMD diagnosis): 52.35 ± 10.49, BMI (kg/m2):24.13 ± 4.07, N Hypertension (%): 62 (51.4)
(10) DEFINE-FMD, USA, N: 126, N Women (%): 126 (100.0), Age (Inclusion): 49.49 ± 11.53, BMI (kg/m2):23.99 + 3.86, N Hypertension (%): NA
(11) UM/CCF-FMD, USA, N: 551, N Women (%): 534 (96.9), Age (Inclusion): 53.77 ± 11.48, Age (FMD diagnosis): 50.29 ± 11.54, BMI (kg/m2):25.13 ± 5.03, N Hypertension (%): 288 (52.2)
(12) UM/CCF-MGI, USA, N: 3436, N Women (%): 3312(96.4), Age (Inclusion): 54.42 ± 12.46, BMI (kg/m2):30.57 ± 9.23, N Hypertension (%): 1512 (44.0)

Recruitment

(1) ARCADIA: FMD patients were included from the ARCADIA (Assessment of Renal and Cervical Artery DysplasIA) register, a national FMD registry at the Georges Pompidou European Hospital, APHP, Paris. The diagnosis of FMD was established using clinical information from the medical history, the interpretation of angiography and/or computed tomography scan of arterial beds after the exclusion of other causes of arterial stenosis such as atherosclerosis, Takayasu disease, Ehlers Danlos syndrome and neurofibromatosis type 1. Given the complexity of the interpretation of imaging of vascular diseases, a local panel of experts including clinicians from the departments of hypertension, radiology, vascular medicine and medical genetics validated the diagnosis.

(2) Three City (3C): The Three-City Study (3C Study) is a population-based longitudinal study of the relation between vascular diseases and dementia in persons aged 65 years and older. Participants were recruited from three French cities: Bordeaux (South-West), Dijon (North-East) and Montpellier (South-East). The 3C Study extended from 1999 to 2012. Participants

underwent regular extensive examination. Examination included measurements of traditional vascular risk factors (blood pressure, glycaemia, lipids, etc.), cognitive functions, and subclinical vascular diseases using carotid ultrasound and cerebral magnetic-resonance imaging (MRI).

(3)Mayo VDB Case-Control study: Potential FMD cases and controls were identified from the Mayo Vascular diseases Biorepository (VDB). Electronic health records of 209 patients including angiographic imaging (computed tomographic, magnetic resonance or catheter based angiography) were manually reviewed by clinicians and the diagnosis was confirmed for 175 patients according to previously reported criteria, of whom 169 (83.4% females) had high-density genotyping data following QC. Controls (n=1141) were participants without known FMD or atherosclerotic vascular diseases.

(4)DEFINE-FMD Study: Eligible cases were females with an imaging-confirmed diagnosis of multifocal FMD and who fulfilled other accepted diagnostic criteria. FMD cases were required to have a clinical diagnosis of multifocal FMD that is confirmed by imaging [computed tomographic angiography (CTA), magnetic resonance angiography, or catheter-based angiography]. Healthy controls were matched to FMD cases according to age and sex, required to be receiving ≤ 2 blood pressure medications, have a body mass index < 35kg/m2 and to be non-smokers. Healthy controls underwent physical exam and those with bruits; unexplained hypertension or other cardiovascular findings were excluded. Exclusion criteria for all subjects included male gender, unifocal FMD, use of immunosuppressive agents, major comorbidities, diseases that may confound genetic/genomic analyses (i.e. Crohn's disease, multiple sclerosis etc.) or any other form of heritable vascular disease (i.e. Ehlers-Danlos, Marfan, Loeys-Dietz).

(5) ARCADIA-POL: Patients were recruited through the ARCADIA-POL (Assessment of Renal and Cervical Artery Dysplasia - POLAND) study, a nation-wide Polish registry for FMD. From January 2015 until December 2018, 343 patients, women and men aged more than 18 years were referred from 32 centers in Poland and were evaluated for suspicion of FMD in the Institute of Cardiology, Warsaw, Poland. FMD lesions in at least one vascular bed were confirmed in 232 patients using whole-body angio-computed tomography. We analysed 129 patients for whom we excluded atherosclerotic stenosis and syndromes where FMD-like lesions are often observed (eg, Ehlers Danlos, Loeys-Dietz).

(6) WOBASZII: We used 298 controls from a randomly ascertained sample from the WOBASZ II (Multicentre National Population Health Examination Survey) study, a population-based Polish cohort7. The WOBASZ II study was planned as a cross-sectional survey of a random sample of Polish residents aged over 20 years. The selection, using the National Identity Card Registry of the Ministry of Internal Affairs, was made as a three-stage sampling, stratified according to administrative units (voivodeships), type of urbanisation (commune), and gender. The study protocol consisted of a questionnaire used in face-to-face interviews, physical examination, and blood samples. WOBASZ II was coordinated by the Department of Epidemiology, Cardiovascular Diseases Prevention and Health Promotion of the Institute of Cardiology in Warsaw in cooperation with medical universities in Gdansk, Katowice, Krakow, Lodz, and Poznan.

(7) University of Michigan/Cleveland Clinic case control study: FMD patients were recruited at the University of Michigan (UM) and/or the Cleveland Clinic. UM/CCF FMD cases were recruited into an IRB-approved study through a referral clinic at the University of Michigan and through self-referral to the study. The Cleveland Clinic cases were enrolled among consecutive patients seen at a dedicated FMD referral clinic. Clinical diagnosis of FMD was ascertained by a vascular medicine specialist after review of diagnostic imaging (computed tomographic, magnetic resonance or catheter based angiography) and prior to blood sample collection. DNAs from healthy controls without vascular disease were obtained from the Cleveland Clinic GeneBank, which was approved by the Cleveland Clinic IRB. Genomic DNA was isolated from a peripheral blood or saliva sample. The Michigan Genomics Initiative (MGI) is a program that recruited participants while awaiting diagnostic, interventional, and surgical procedures. Participants provided a blood sample for genetic analysis and agreed to link their sample to their electronic health record and other sources of health information. Several ICD codes corresponding to diagnoses of arterial aneurysm, dissection, and non-atherosclerotic dysplasia and stenosis were excluded, as well as connective tissue disorders, as previously described. The hypertension variable was defined as any ICD code containing the "hypertension" term for MGI controls.

(8) FMD European/International Registry and Initiative (FEIRI): Patients were enrolled through the European/International FMD Registry and Initiative (FEIRI). FMD was defined as the presence of an idiopathic, segmental, nonatherosclerotic, and noninflammatory stenosis (either with focal or string-of-beads appearance) of a small- or medium-sized artery in at least 1 vascular bed, documented with computed tomographic angiography, magnetic resonance angiography, or catheter-based angiography imaging. Patients with a suspicion of FMD only based on duplex ultrasound were excluded. Patients whose primary diagnosis was spontaneous coronary artery dissection were not eligible, even in the presence of extracoronary FMD lesions. Data on demographic and FMD characteristics were collected through the FMD Registry.

(9) ASKLEPIOS: The Asklepios Study is a longitudinal population study focusing on the interplay between ageing, cardiovascular haemodynamics and inflammation in preclinical cardiovascular disease11. Participants aged 35 to 55 years were sampled from the twinned Belgian communities of Erpe-Mere and Nieuwerkerken. Exclusion criteria were the presence of atherosclerotic lesions and concomitance with major illness, diabetes or conditions precluding accurate haemodynamic assessment. All participants underwent systematic physical examination during a continuous 2-year period, between October 2002 and September 2004 at a single study site in Erpe–Mere involving: measurement of basic clinical data, blood samples collection, echocardiographic examination and vascular echography and tonometric measurements.

(10) FMD cohorts may suffer from an enrichment of patients presenting with phenotypes such as hypertension or stroke compared to an hypothetical FMD population. Indeed, investigation for FMD phenotype involves semi-invasive approaches that are generally not justified if patients do not present major phenotypes. For example, an important fraction of FMD patients in European studies were identified following referal hypertension centers of excellence. We therefore used several independent approaches (adjustment and stratification on hypertension status when it was possible, conditioning on SBP genetic association) to confirm that hypertension or more largely blood pressure regulation was not a confounding factor for the associations of FMD top loci. In addition, the meta-analysis should remove part of this potential bias as cohorts followed different referral precedures. However we can not exclude that the observed genetic correlations between FMD and other

vascular traits and diseases may be impacted by this potential bias, as we mentionned in the discussion.

Ethics oversight	(1) ARCADIA/PROFILE protocol was approved by the Ile-De-France research ethics committee (Comité de Protection des Personnes: CPP d'île de France) on 03/04/2009 (ID: 2009-A00288-49).<br><br>(2) The Three-City Study (3C Study) was approved by "comité consultatif de protection des personnes dans la recherche biomédicale Bicêtre Hôpital Bicêtre n°99-28 CCPPRB approved 10/06/99, 11/03/2003 and 17/03/2006.<br><br>(3) Mayo VDB Case-Control study was approved by the Mayo Clinic Institutional Review Boards (IRB # 08–008355).<br><br>(4) DEFINE-FMD study was approved by the Human Research Ethics Committee of the Icahn School of Medicine at Mount Sinai (Study ID: HS#13-00575/GCO#13–1118 and is registered with ClinicalTrials.gov Identifier: NCT01967511.<br><br>(5) ARCADIA-Pol study was approved by Local Ethics Comittee, Institute of Cardiology, IK-NPIA-0021017/1482/17.<br><br>(6) WOBASZII study was accepted by the Field Bioethics Committee of the Institute of Cardiology in Warsaw.<br><br>(7) The UM/Cleveland case control study was approved by each institution IRB protocols: The University of Michigan IRB number HUM00044507 and the Cleveland Clinic IRB number 10–318.<br><br>(8) All centres included in FEIRI received approval from the respective local/ national ethics committees.<br><br>(9) The ethical committee of the Ghent University Hospital approved ASKLEPIOS study protocol.

Note that full information on the approval of the study protocol must also be provided in the manuscript.
